# A Data-Driven Framework for Direct Local Tensile Property Prediction of Laser Powder Bed Fusion Parts

**DOI:** 10.3390/ma16237293

**Published:** 2023-11-23

**Authors:** Luke Scime, Chase Joslin, David A. Collins, Michael Sprayberry, Alka Singh, William Halsey, Ryan Duncan, Zackary Snow, Ryan Dehoff, Vincent Paquit

**Affiliations:** 1 Electrification and Energy Infrastructure Division, Oak Ridge National Laboratory, Oak Ridge, TN 37830, USA; sprayberryma@ornl.gov (M.S.); singhar@ornl.gov (A.S.); halseywh@ornl.gov (W.H.); snowzk@ornl.gov (Z.S.); paquitvc@ornl.gov (V.P.); 2 Manufacturing Science Division, Oak Ridge National Laboratory, Oak Ridge, TN 37830, USA; joslincb@ornl.gov (C.J.); duncanrk@ornl.gov (R.D.); dehoffrr@ornl.gov (R.D.); 3 Materials Science and Technology Division, Oak Ridge National Laboratory, Oak Ridge, TN 37830, USA; collinsda@ornl.gov

**Keywords:** laser powder bed fusion, tensile properties, machine learning, in situ monitoring

## Abstract

This article proposes a generalizable, data-driven framework for qualifying laser powder bed fusion additively manufactured parts using part-specific in situ data, including powder bed imaging, machine health sensors, and laser scan paths. To achieve part qualification without relying solely on statistical processes or feedstock control, a sequence of machine learning models was trained on 6299 tensile specimens to locally predict the tensile properties of stainless-steel parts based on fused multi-modal in situ sensor data and a priori information. A cyberphysical infrastructure enabled the robust spatial tracking of individual specimens, and computer vision techniques registered the ground truth tensile measurements to the in situ data. The co-registered 230 GB dataset used in this work has been publicly released and is available as a set of HDF5 files. The extensive training data requirements and wide range of size scales were addressed by combining deep learning, machine learning, and feature engineering algorithms in a relay. The trained models demonstrated a 61% error reduction in ultimate tensile strength predictions relative to estimates made without any in situ information. Lessons learned and potential improvements to the sensors and mechanical testing procedure are discussed.

## 1. Introduction

As a new class of manufacturing processes, metal additive manufacturing (AM) [1] holds significant promise for the rapid production of small-to-medium quantities of parts with complex geometries and internal structures [2]. For industries producing safety-critical components, part qualification is an integral step of any manufacturing process [3]. Qualification frameworks for traditionally manufactured components typically fall into one or more of the following paradigms: (1) destructive or nondestructive testing of a representative sample of the larger population of manufactured components or of designated coupons to detect process drift [4] (i.e., statistical quality control), (2) maintenance of a robustly defined, in-control manufacturing process [5] (i.e., process qualification) combined with a set of materials specifications for the feedstock or workpiece [6] (e.g., usage of A- and B-basis allowables), and (3) non-destructive evaluation (NDE) [7,8] of the entire population of manufactured components (i.e., part-specific qualification). Because these traditional qualification approaches are often incompatible with additively manufactured components, this work proposes a data-driven qualification framework that leverages in situ data to directly predict localized static tensile properties.

Laser-powder bed fusion (L-PBF) is a widely adopted metal AM process in which a laser beam is used to melt powder feedstock into a stack of two-dimensional 2D cross-sections of the intended part geometry [1,2]. Many current and anticipated business cases for L-PBF components [2] are not compatible with traditional qualification paradigms. For example, because one advantage of AM is its ability to make small production runs of customized designs [2], many L-PBF designs are not produced in quantities conducive to population-based qualification. Furthermore, as articulated by Seifi et al. [3], L-PBF’s relative novelty, geometry-dependent process dynamics, and the nature of localized re-solidification of the feedstock challenges many of the assumptions required to qualify the process and materials in a way that is agnostic to part geometry. Finally, components optimized for L-PBF often have complex internal geometries [2], rough surfaces [9], or are manufactured with high atomic number alloys [10], complicating traditional post-build NDE techniques [8].

Fortunately, the layer-wise nature of L-PBF, along with the incremental re-solidification of discrete volumes within a layer, provide unique opportunities for in situ process monitoring [11] because each sub-volume is directly observable at some point during the manufacturing process. This facet of AM offers a significant advantage over many NDE techniques, which struggle to spatially resolve flaws in three dimensions, particularly for complex geometries. Such in situ data can also be used to construct a *component digital twin* [12] of individual components. As defined by Grieves and Vickers [13], a component digital twin is a virtual copy of its *physical twin*—a specific instance of a manufactured component that can be simulated [14]. This work develops new models to convert in situ data into localized material property predictions that could be used to instantiate a component digital twin.

Critically, properties predicted by these models should be localized (i.e., valid for a sub-volume of a component) so the models can be generalized to arbitrary component geometries. Scime et al. [15] proposed combining deep learning (DL), machine learning (ML) [16], and feature engineering [17] algorithms in an augmented intelligence relay (AIR) such that each algorithm solves a sub-problem within the overall data workflow. Over the last decade, the AM in situ process monitoring community has made significant strides in applying signal processing and computer vision techniques to both temporal [18,19,20,21,22] and spatial [23,24,25,26,27] data for the purposes of anomaly and flaw detection. A growing number of researchers are studying co-registered multi-modal sensor data stacks [28,29] and leveraging DL algorithms to achieve pixel-wise anomaly and flaw segmentation [28,30,31]. Although rarely generalized or presented in the context of a larger qualification framework, visualization of in situ data [32], including its registration to ex situ measurements [33,34], is also relatively common in the literature. In contrast, relatively little work has been reported regarding direct property prediction for localized sub-volumes, which is the focus of this work.

Most tensile property measurement and prediction research reported in the literature [35] for metal AM is similar to that of Lavery et al. [36], which correlates tensile measurements to laser processing parameters, post-build treatments such as hot isostatic pressing (HIP), and porosity content measured post-build. Similarly, Kusano et al. [37] extracted microstructural features from scanning electron microscopy (SEM) images of L-PBF-processed Ti-6Al-4V and performed multiple linear regression to fit tensile property prediction models to these feature vectors. Thematically similar, Hayes et al. [38] used constitutive modeling to predict the yield strength of Ti-6Al-4V processed via directed energy deposition (DED) based on microstructural features measured post-build. A related area of research garnering significant attention is the prediction of fatigue life based on flaw populations measured post-build using either x-ray computed tomography (XCT) of the part or destructive microscopy of witness coupons. In one example of this approach, Romano et al. [39] developed analytical models capable of predicting the fatigue properties of L-PBF-processed AlSi10Mg.

When considering only studies that use in situ sensor data to inform material property prediction models, the authors determined that most existing work has focused on polymer fused deposition modeling (FDM) processes, such as that performed by Zhang et al. [40]. Interestingly, Seifi et al. [41] performed DL analyses of melt pool thermal images to detect flaws in DED-processed Ti-6Al-4V. The resulting data were then used to inform traditional fatigue life models. Importantly, this approach required not only detecting the presence and location of each flaw but also estimating their size. Bisht et al. [42] correlated the relative occurrence of anomalies observed in on-axis photodiode data with the measured plastic elongation of L-PBF-processed Ti-6Al-4V, thus demonstrating a potentially viable approach that jumps directly from in situ data processing to localized property predictions. Finally, among the most similar work is that of Xie et al. [43], which encoded in situ thermal history measurements into engineered features and predicted local tensile properties for thin-walled Inconel 718 DED components using a neural network trained on tensile data from extracted tensile specimens.

This work uses a relay of machine-learned algorithms (Section 2.1) to predict local static tensile properties based on in situ data for L-PBF components. Computer vision techniques were used to process the in situ sensor data and to spatially register it to the ex situ mechanical test results (Section 2.2). Localized property prediction required a bespoke build strategy (Section 2.6) and drove many of the decisions regarding specimen geometry and post-processing methodologies. An extensive cyberphysical infrastructure was implemented to enable robust spatial tracking of thousands of individual tensile specimens (Section 2.7) and facilitate the public availability of the entire 230 GB Peregrine v2023-11 dataset [44] used in this research. Neural networks were designed to first segment anomalies apparent in the in situ sensor data (Section 2.3) and then to predict local tensile properties based on human-engineered feature vectors (Section 2.10). Model validation and testing performance results are presented in Section 3.1 and Section 3.2, respectively. Ultimately, the goal of the proposed approach is to support future qualification paradigms that rely more heavily on the standardization of in situ sensor suites and validated algorithms and less heavily on certification of “locked-down” manufacturing processes and material specifications.

## 2. Materials and Methods

### 2.1. Experimental Conditions and Data Analysis Framework

Experiments were performed at the Manufacturing Demonstration Facility (MDF) located at Oak Ridge National Laboratory (ORNL) in Oak Ridge, Tennessee. Specimens were printed using stainless-steel (SS) 316L powder on a Concept Laser M2 (General Electric, General Electric Additive, Cincinnati, OH, USA) L-PBF printer. The Concept Laser M2 had two 400 W laser modules with overlapping fields of operation, and a compliant recoater blade was used to spread the powder. Algorithms were developed in Python v3.7 with relevant dependencies, including TensorFlow v1.13.1, OpenCV v3.4.1, and Scikit-image v0.18.1. Computations were benchmarked on a desktop computer equipped with two Quadro RTX 5000 (Nvidia Corporation, Santa Clara, CA, USA) graphical processing cards, two 16-core 2.10 GHz processors, and 256 GB of volatile memory. The AM terminology used in this document complies with ISO/ASTM 52900:2015 [1] where appropriate.

Because in situ data from L-PBF processes contain complex, multi-modal, contextually dependent information [28,45], it is not easily interpreted solely by physics-based models or human-designed heuristics. Therefore, the authors propose that machine-learned models are the most viable approach for translating in situ data to localized property predictions. Decomposing the data workflow into a relay enabled the use of both ML and DL models, even when the ground truth tensile properties were expensive to collect. This is possible because physics-informed decisions could be made at each interface throughout the relay to reduce the complexity of the feature encodings, which must be learned from the experimental data. For example, features encoding the laser scan vector length within a sub-volume were explicitly designed rather than learned based on the a priori knowledge that the scan length might affect solidification conditions [46] and, therefore, local material properties. The use of a relay provided other advantages, including (1) computationally efficient translation of data across spatial size scales, (2) improved interpretability of property predictions, (3) improved model generalizability, and (4) increased opportunities for modularity within the framework.

**Figure 1:**
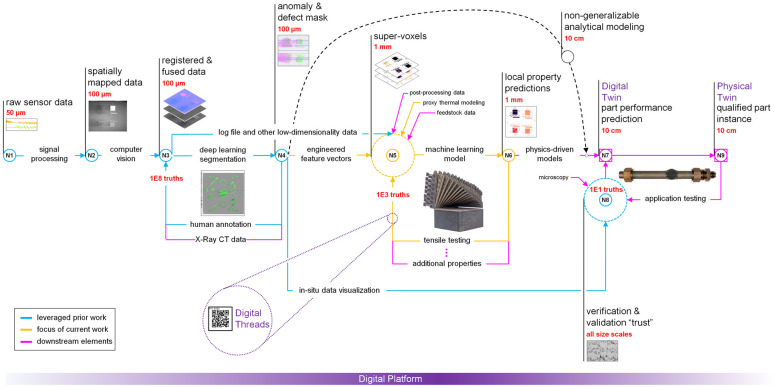
Graphical representation of the implemented AIR. This paper focuses on nodes N5 and N6 (orange), leveraging prior work by the authors shown at nodes N1, N2, N3, N4, and N8 (blue). Approximate spatial resolutions of data at each node are shown in red. Approximations for the number of ground truth values required for each feedback loop are also shown in red. Key elements of the digital platform that supported the implementation of the relay are shown in purple. The black dashed line from N4 to N7 represents a possible alternate pathway for predicting localized properties.

Figure 1 depicts the implemented AIR, including elements developed in prior work (blue) and downstream elements (magenta), which are beyond the scope of this paper. Starting at N1, natively temporal data (i.e., laser scan order) were spatially mapped as rasterized images for each print layer, while natively spatial data (i.e., visible-light images of the powder bed) entered the AIR directly at N2. At N3, the in situ data streams and design intent information (i.e., part geometry) were co-registered and placed into a common coordinate system. At N4, a subset of the fused sensor data was processed by a DL image segmentation model to produce an anomaly mask for each print layer. Training this DL model (feedback loop between N4 and N3) occurred pixel-wise using approximately 180 million ground-truth classifications acquired via expert annotation [28]. Up to this point in the relay, the spatial resolution of the data was on the order of 100 µm. At N5, the print volume was demarcated into 1 mm *super-voxels*, defined here as a sub-volume of a component for which local material property predictions can be made by the AIR. Associated with each super-voxel are (1) an engineered feature vector encoding the in situ sensor data, (2) anomaly segmentation metrics, (3) part geometry, and (4) proxy representations of the local thermal history. Finally, an ML model was trained (feedback loop between N6 and N5) using 6299 tensile tests to predict the local tensile properties at N6 based on the super-voxel feature vectors. The cyberphysical infrastructure that supported the AIR is referred to as the *digital platform*, and it allowed the in situ and ex situ data to be tracked as *digital threads* [15].

While beyond the scope of this work, downstream elements of the AIR should be considered to understand how the current research fits into the ultimate goal of achieving part-specific qualification. For example, a component digital twin consisting of a canonical finite element model of the component could be instantiated at N7 using the local material properties predicted at N6. This contrasts with traditional finite element meshes, which typically use material properties derived from a statistical analysis of aggregated historical data.

### 2.2. Multi-Modal Data Collection, Co-Registration, and Fusion

**Figure 2:**
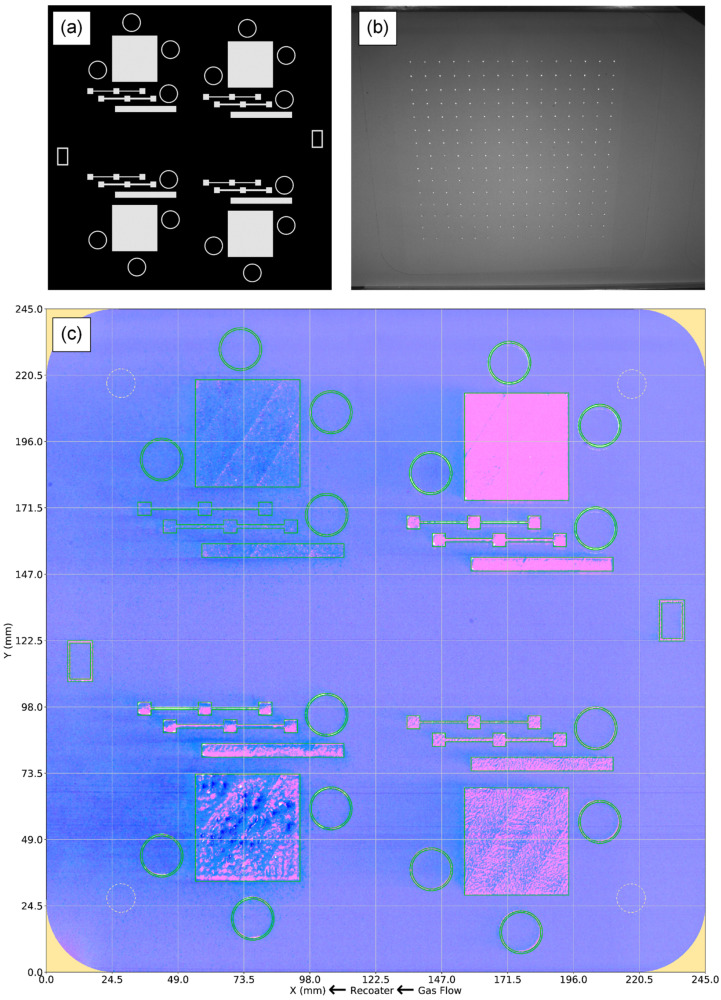
(**a**) A binary image representing the nominal CAD geometry (white) for layer 650 (32.50 mm) of build B1.2. (**b**) A raw, post-melt, QM coating layer image showing the printed calibration cone grid. The raw images are affected by perspective and lens distortions and suffer from uneven lighting across the print area. (**c**) A false-color image fusing the calibrated post-melt and post-spreading visible-light images from layer 650 (32.50 mm) of build B1.2. The nominal CAD geometry is indicated by the green outlines, and typical “keep-out” regions of the print area are marked in beige. Arrows indicate recoating and shield gas flow directions. The dynamic range of the composite image has been modified to accentuate features of interest, such as the horizontal streaks from the recoating mechanism.

To enable local tensile property predictions, data from multiple in situ and a priori sources were spatially mapped (N2) and co-registered (N3) within a common coordinate system defined by the computer-aided design (CAD) model of each build. The CAD model was first converted into the standard triangle language (STL) file format and sliced using the Magics Image Build Processor (Materialise, Leuven, Belgium) into a set of binary layer images (Figure 2a). Each binary image was globally thresholded to identify the locations of the printed geometries, and a two-pass, 2.5D implementation of a standard 2D connected-components algorithm [47] was performed to uniquely segment each printed part within the 3D build volume. Each component was automatically assigned an identifier based on its position within the 3D build volume, and this information was shared with the digital platform, which generated a globally unique identifier. The universal coordinate system was defined in reference to ISO/ASTM 52900:2015 [1], with the positive *x*-axis oriented left–right from the perspective of the printer operator, the positive *y*-axis oriented front–back, and the positive *z*-axis oriented vertically along the build direction. The powder recoating and shield gas flow directions are parallel to the negative *x*-axis, as shown in Figure 2c.

The original equipment manufacturer (OEM) quality monitoring (QM) coating [48] camera captured two visible-light 5 mega-pixel (MP) images during each layer: one after melting was complete and one after powder had been spread across the print bed. Because this camera was mounted at an angle relative to the normal vector of the print bed, distorted images were produced. To correct this distortion and transform the image data into the common reference frame, a calibration pattern consisting of a 15 × 15 grid of cones with a 12 mm center-to-center spacing was printed and imaged (Figure 2b). The planar center of each cone was automatically detected and used to calculate a homographic transformation matrix using the least median of squares (LMedS) [49] method implemented by OpenCV. To increase the contrast between the printed dots and the surrounding powder and to facilitate the automatic detection of their centroids, multiple consecutive post-melt images were background-subtracted, and their difference images were composited together. After Gaussian blurring to further reduce noise, Otsu’s method [50] was used to threshold the dot grid image. A connected-components analysis of the binary image enabled outlier dots to be rejected based on size, aspect ratio, areal fill, and center-to-center distance criteria. Finally, a bounding rectangle was fit to the dot grid to identify the four outer corner dots, and each dot was matched to its presumed corresponding dot in the target dot pattern.

The transformed image was then cropped to a physical size of 245 × 245 mm, which encompassed the printable area of the Concept Laser M2 and produced a calibrated image size of 1842 × 1842 pixels with a spatial resolution of approximately 130 µm. The resolving power of the camera setup ranged from 220 to 280 µm across the powder bed, as measured using a USAF 1951 camera resolution target (Edmund Optics, Barrington, NJ, USA). The registration error between the CAD geometries and the imaging data was estimated at approximately 250 µm. To compensate for uneven lighting conditions over the print area, a smoothed image of an anomaly-free powder spread was used to generate a lighting correction mask. Figure 2c shows a fused representation of the two visible-light images after the perspective and lighting corrections have been applied, along with the nominal CAD geometry. Unless otherwise specified, all following images of the print area are in the same coordinate system and have the same field of view as introduced in Figure 2c.

**Figure 3:**
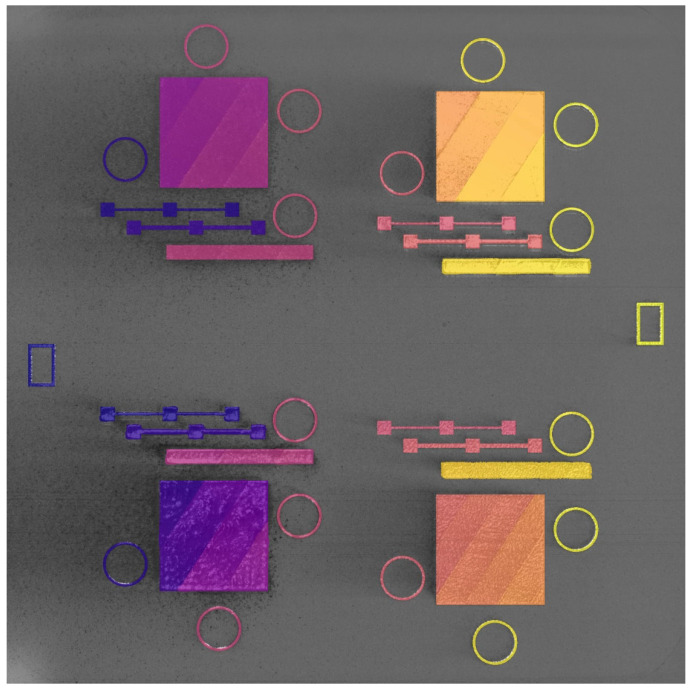
A false-color image fusing the laser scan path information with the calibrated post-melt visible-light image. The color map represents the time since the start of the layer, with darker blue regions melted first and lighter yellow regions melted last. The laser stripe boundaries are clearly visible as diagonal discontinuities in the color map. These data are from layer 650 (32.50 mm) of build B1.2.

Unlike the inherently spatial visible-light images, the OEM log file recorded machine health metrics temporally at sampling rates on the order of 5 Hz. The temporal log file values used included (1) the total layer time measured in seconds, (2) the top and (3) bottom argon flow rates measured in cubic meters per hour, (4) the oxygen percentage within the build chamber, (5) the temperatures of the build plate and (6) bottom argon gas flow measured in degrees Celsius, and (7) the gas ventilator flow rate measured in cubic meters per hour. For the purposes of this work, these low-frequency temporal data were spatially mapped by assigning the average of the values recorded over the duration of a given print layer to that print layer. Laser scan path information was recovered from the OEM QM Meltpool [48] system, which records laser location data at approximately 40 kHz in a technical data management streaming (TDMS) file format (National Instruments, Austin, TX, USA). Spatial mapping and registration of laser scan order data were achieved using the methods described by Halsey et al. [51]. However, in the previously reported implementation, on-axis photodiode data were used to filter out the “skywriting” that occurs at the laser beam turnaround points, as well as the “jump lines” between printed components. To improve the reliability of this artifact removal process for this effort, skywriting detection was instead performed using the laser travel vector to detect the turnaround points, as well as an empirically derived travel duration to identify the surrounding turnaround region. Similarly, jump lines were detected based on an empirically derived laser speed threshold. Figure 3 shows a visualization of the laser scan path within a single layer of a build. The QM Meltpool data used included the laser module and the laser scan path. Data from the QM Meltpool on-axis photodiodes and melt pool cameras were not available for this specific printer.

### 2.3. Powder Bed Anomaly Segmentation and Training Methodologies

While the CAD geometries, log file data, and laser scan path information were directly incorporated into the super-voxels at N5, the two visible-light layer images were first processed by a dynamic segmentation convolutional neural network (DSCNN) DL algorithm at N4. The DSCNN converted the high-dimensional multi-modal image data into a lower-dimensional embedding that encoded salient features across multiple size scales in a latent space. That is, the DSCNN performed a semantic segmentation [52] task and produced pixel-wise classifications of various anomalous and nominal L-PBF conditions. Because the DSCNN architecture was previously reported by Scime et al. [28], only the germane differences in its implementation are noted here.

**Figure 4:**
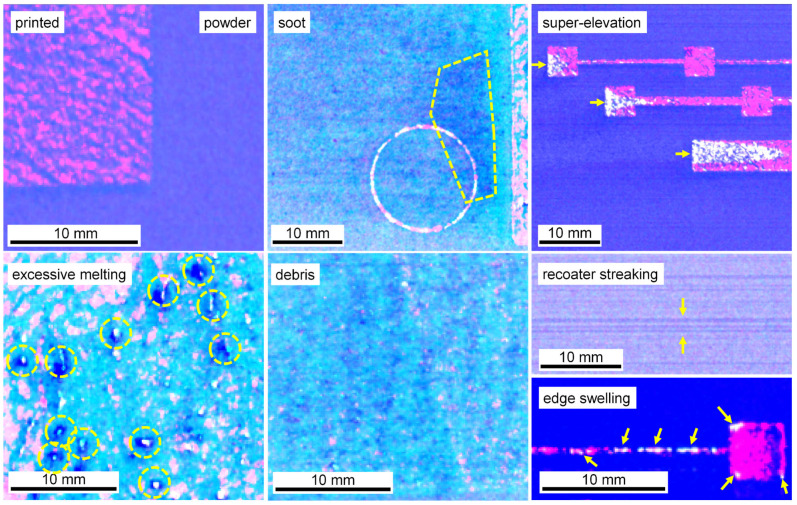
False-color images fusing the post-melt and post-spreading visible-light images of each of the eight powder bed classes that were segmented by the DSCNN. The dynamic range of each image has been modified to accentuate features of interest. Annotations are presented in yellow to highlight the relevant sensor indications. Some classes have well-defined boundaries, such as edge swelling and excessive melting, while others have nebulous boundaries, such as soot and debris, and are delineated as entire regions during annotation.

Prior work demonstrated the DSCNN’s ability to classify a variety of powder bed anomaly classes across multiple powder bed printer types [28]. Eight classes were identified as potentially relevant to tensile property prediction, examples of which are shown in Figure 4. The *powder* and *printed* classes represent the two nominal L-PBF conditions, respectively, indicating the unfused and fused material. *Recoater streaking* generally occurs when the recoating mechanism is either damaged or dragging debris across the powder bed and is visually characterized as a long streak parallel to the recoating direction [53]. *Edge swelling* appears as small regions of the part, typically corners and edges, elevating above the spread powder layer. Although edge swelling is common in L-PBF, even under nominal processing conditions, certain process parameter changes and local part geometries can increase its occurrence [54,55]. *Debris*, in the context of this work, generally indicates low energy density melt parameters prone to causing lack-of-fusion porosity [56]. *Super-elevation* of large regions of the part above the powder layer can occur either as the result of warping caused by residual thermal stresses [57] or improper powder dosing. *Soot* refers to spatter particles [58] that have landed on the powder bed. For the imaging system used in this work, soot generally manifests as clouds of dark particles. In the context of this work, *excessive melting* indicates high energy density melt parameters prone to causing keyhole porosity [59] and is visually characterized and labeled by a bubbling of the part surface.

Ground truth training data for the DSCNN were collected through expert annotation of 180 million pixels across 38 print layers sourced from 23 different Concept Laser M2 builds, all using SS 316L feedstock. For this task, annotators leveraged a purpose-designed graphical interface incorporating image calibration, data fusion, dynamic range scaling, standard drawing tools (e.g., brushes, lassos, flood fills), intensity thresholding options, and ML-assisted annotation capabilities. While it is also possible to train the DSCNN using ground truths collected from ex situ characterization (e.g., flaw detections from XCT), this approach is beyond the scope of this work. The manual annotation procedure is further described in Scime et al. [28], and example annotations and associated data can be downloaded from the Peregrine v2021-03 [60] dataset.

Data augmentation was used to increase the size of the training set without requiring the collection of additional ground truths [61]. The augmentation mechanisms applied during training differed slightly from those reported in Scime et al. [28]; they consisted of (1) global intensity shifts with magnitudes uniformly distributed between −15% and +15% of the dynamic range of each image channel, (2) additive Gaussian noise distributions with variances equal to 0.0001% and 0.001% of the dynamic range of each image channel, and, new in this work, (3) spatial shifts of each image tile by up to 20 pixels in each direction. The spatial shift augmentation technique was included to increase the total amount of training data and to reduce artifacts at the edges of the tiles. The DSCNN was trained with a cross-entropy loss function weighted by the inverse of the class frequencies as specified by Equation (6) in Scime et al. [28]. Additional pixel-wise weights were applied to disincentivize the optimization function from spatially expanding less common classes (e.g., edge swelling) at the expense of more common classes. This re-balancing was achieved by increasing the weight of the ground truths for powder and printed pixels located near an interface with a less common class. This weight adjustment, w, is given by Equations (1)–(3).

(1) $$m=\frac{MIN\left(0,\frac{N_{all}}{N_{int}}\right)-1}{\phi },$$

(2) b=1−m,

where ϕ is a saturation distance set at 1% of the image size, $N_{all}$ is the total number of pixels in the image, and $N_{int}$ is the number of pixels closer to an interface than the saturation distance.

(3) $$w\left(i,j\right)=m\left[MAX\left(1,\phi -{[D}_{1}\cup D_{2}]\left(i,j\right)\right)\right]+b,$$

where $w\left(i,j\right)$ is the interfacial weight adjustment at pixel $\left(i,j\right),$ and $D_{1}$ and $D_{2}$ are the distance transforms [62] for the combined powder and printed annotation masks.

Other noteworthy changes from [28] include the preservation of the native bit depth (16 bits) of the visible-light images and the implementation of training early-stop [63] criteria based on detecting a plateau in the epochal validation accuracy. Additionally, two heuristics were applied to the segmentation mask produced by the trained DSCNN. The first heuristic converted excessive melting segmentations further than 750 µm from the CAD geometry to debris. This mitigates observed confusion between these classes and is justifiable because excessive melting definitionally cannot occur beyond a melted part. The second heuristic extends recoater streaking segmentations horizontally across the print area. This is necessary because, although recoater streaking is readily apparent in the unfused powder, it is generally difficult to observe over the top of a printed part. However, the literature suggests that L-PBF recoater streaks often extend into the parts themselves [34], especially when a compliant recoater is used. Specifically, this heuristic is triggered by DSCNN recoater streaking segmentations with horizontal dimensions larger than 5 mm, and only pixels initially classified by the DSCNN as either powder or printed material are overwritten by the heuristic.

### 2.4. Performance of the Dynamic Segmentation Convolutional Neural Network

The overall performance of the DSCNN architecture is extensively documented in Scime et al. [28]. Table 1 reports the true-positive (TP) and false-positive (FP) validation rates for the specific DSCNN model and training dataset used for the AIR. The significant TP performance improvements (e.g., from 17.5% to 85.8% for soot) relative to those reported by Scime et al. [28] are the result of the increased size of the training database and the modified training procedures described above.

**Figure 5:**
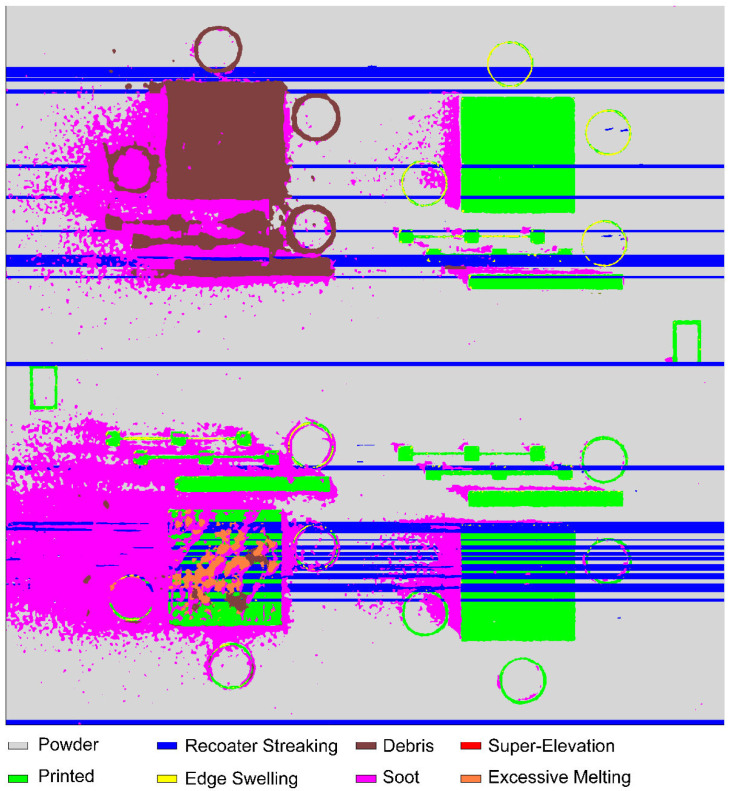
Pixel-wise segmentation results from the trained DSCNN for layer 650 (32.50 mm) from build B1.2. Anomaly classes are indicated by color.

Figure 5 shows an example of a segmented layer. Note that while heuristics were enabled for Figure 5, they are not included in the validation metrics. The average DSCNN inference time is 1.7 s, and the layer-wise connected-component analysis of the CAD geometry is typically less than 2 s, depending on the geometry. Loading the visible-light images into computer memory and performing the image calibration procedure may be performed in parallel with DSCNN inference.

### 2.5. Specimen Design

**Figure 6:**
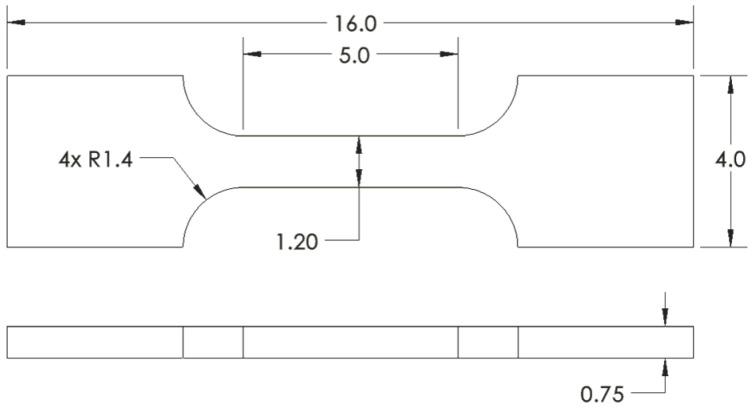
Key nominal dimensions of the SS-J3 tensile specimens. All values are in millimeters.

Four factors influenced the mechanical specimen design: (1) the material properties of interest, (2) the importance of measuring representative material properties across multiple as-printed part geometries, (3) the compatibility of the physical specimen size with the computational constraints of the models, and (4) adherence to accepted material characterization standards. First, tensile properties, including yield strength (YS), ultimate tensile strength (UTS), uniform elongation (UE), and total elongation (TE), were selected as prediction targets for this work. Whereas other material properties, such as fatigue life, are expected to be comparatively more sensitive to processing anomalies [39], the shorter testing cycles for room temperature static tensile tests enabled the collection of many more ground truths. Additionally, static tensile properties were of interest for nuclear power applications [64], and there is sufficient literature indicating that variations in L-PBF processing conditions (e.g., geometric feature size, solidification conditions, and void-type flaws) could induce variations in these properties [36,65,66,67,68]. Next, to maintain the generalizability of the ML models to multiple component geometries and local thermal histories, the tensile specimens were extracted from a set of larger as-printed geometries instead of being printed in their final shape, as has been conducted in prior high-volume tensile testing work by Roach et al. [65]. For clarity, specimens extracted from as-printed *parts* will be referred to as *samples* throughout the remainder of this manuscript. Because the spatial resolution of the ground truth tensile data was controlled by the size of the specimen’s gauge section, the standard [69] subsize SS-J3 geometry shown in Figure 6 was selected, as described in Appendix A.

**Figure 7:**
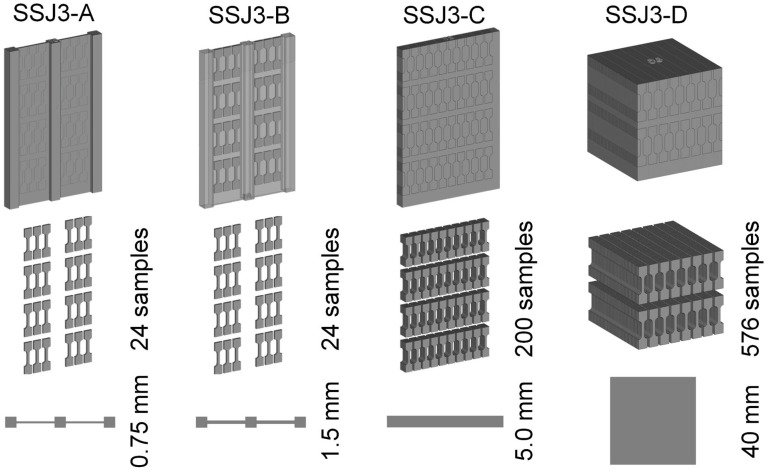
An isometric view of the CAD model for each of the as-printed SSJ3 part geometries (**top row**), the 3D layouts of the SS-J3 samples relative to their corresponding printed part volumes (**middle row**), and a top-down view of the part geometries (**bottom row**). The number of potential samples and the nominal wall thickness for each geometry are given in the middle and bottom rows, respectively.

The SS-J3 samples were extracted from four different printed part geometries designated as SSJ3-A, SSJ3-B, SSJ3-C, and SSJ3-D, as indicated in Figure 7. The CAD models of each geometry were adjusted to achieve as-printed dimensions as close as possible to the reported nominal dimensions. The SS-J3 samples were distributed along the nominal build direction with vertical center-to-center spacings of 19 mm. The SSJ3-A and SSJ3-B geometries incorporated buttresses to increase part stiffness and maintain the dimensional accuracy of the parts during printing, heat treatment, and machining. Note that while the SSJ3-C and SSJ3-D parts produced samples with as-printed and machined surfaces, the SSJ3-A parts produced samples with only as-printed surfaces, and the SSJ3-B parts produced samples with only machined surfaces.

### 2.6. Build Design and Conditions

For all builds in this work, the nominal print layer thickness was maintained at 50 µm, no preheating was performed, and argon was used as the shield gas. The feedstock was TruForm (Praxair Surface Technologies, Indianapolis, IN, USA) SS 316L powder sourced from a single lot. The manufacturer reported D10, D50, and D90 powder particle diameter values of 20 µm, 31 µm, and 43 µm, respectively. The chemical composition of the virgin powder, as reported by the manufacturer, is provided in Table 2.

The bulk regions of the SS-J3 samples were melted using the laser raster process parameter sets defined in Table 3. The *nominal* parameter set was provided as the default for SS 316L by Concept Laser, the *best* parameter was chosen to minimize porosity, the *lack-of-fusion* parameter was known to produce significant lack-of-fusion (*LOF*) porosities, and the *keyhole* parameter was chosen to induce keyholing pores. It was expected that the varying energy densities and solidification cooling rates of these parameter sets would result in substantially different as-printed microstructures and void-type flaw populations, as shown in other work [67]. Table 3 also defines the *soot* parameter, which did not directly melt any SS-J3 samples but was instead used to generate abnormally large quantities of soot near some of the tensile samples, with the goal of introducing spatter-induced porosities as observed by Schwerz et al. [23] and others. The default post-contour parameter set provided by Concept Laser was applied with a laser beam power of 120 W, a laser beam speed of 220 mm/s, and a laser spot size of 70 µm.

A total of five L-PBF builds were performed to generate 6299 SS-J3 tensile specimens used in the feedback loop connecting N6 and N5. Additional builds were performed for camera calibration, algorithm development, DSCNN training, process parameter development, specimen design, heat treatment development, and specimen extraction and tracking procedure development as described in [70]. Design requirements for the five builds discussed in this work include (1) facilitating the extraction of thousands of SS-J3 tensile specimens from trackable locations, (2) capturing the range of process and part variability expected to occur during L-PBF manufacturing, and (3) generating a range of local tensile properties caused by various mechanisms hypothesized to correlate to signatures observable in the available in situ sensor data. The number SS-J3 samples extracted from each build and the variable build conditions which were expected to result in variable tensile properties are summarized in Table 4.

**Figure 8:**
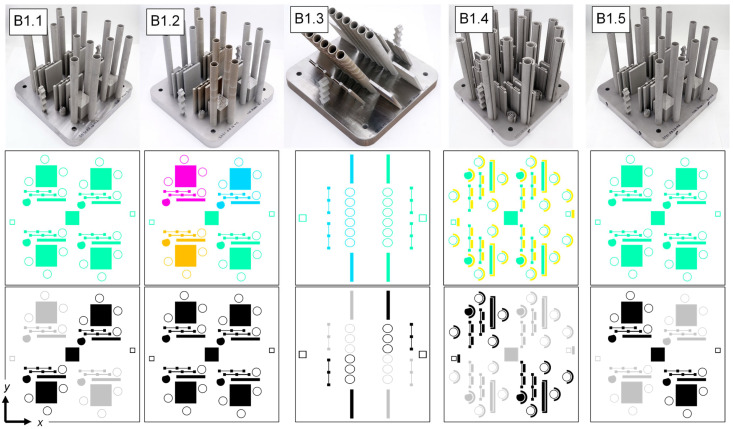
Each column shows the build layout for a given build. The top row contains isometric views of each build after the print was completed. The middle row colors each part by the process parameter set, with nominal, best, LOF, keyhole, and soot indicated by green, blue, purple, orange, and yellow, respectively. The bottom row colors each part by the laser module, with the first laser module indicated by black and the second laser module indicated by gray. Note that the yellow, soot-generating parts in B1.4 are located upstream of the gas flow relative to the SS-J3 samples.

Figure 8 shows an isometric view of each build, along with the part layout, process parameter assignments, and laser module assignments. At a high level, B1.1 was designed to produce baseline process conditions, B1.2 was designed to capture the effects of variable laser raster processing parameters, B1.3 was designed to represent overhanging geometries, B1.4 was designed to capture the effects of spatter particles and decreased argon gas flow, and B1.5 was designed to capture the effects of recoater blade damage and powder short feeds. Intermediate visualizations (N8) of the in situ data and DSCNN segmentation results for each build are provided in Appendix B for additional context.

### 2.7. Sample Extraction and Tracking

The printed parts were first heat-treated while attached to their build plates to relieve residual thermal stresses induced during printing [57]. Relieving these stresses reduced the amount of distortion experienced by the parts during sample extraction, improving both the dimensional tolerances of the specimens and the fidelity of the registration between the tensile test results and the in situ data. Full heat treatment details are provided in Appendix C.

**Figure 9:**
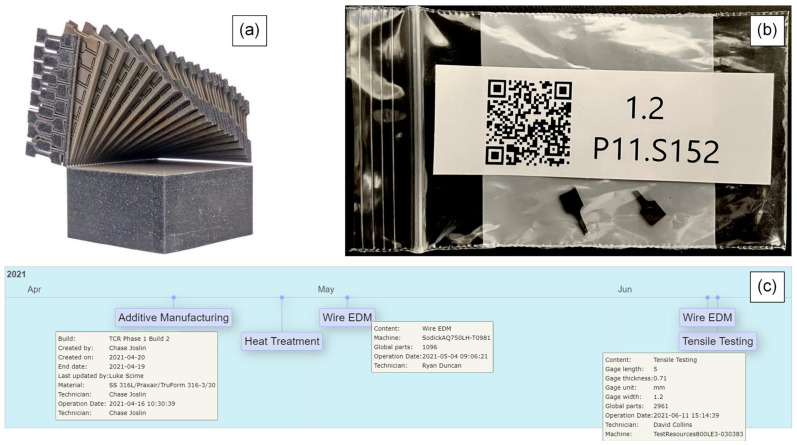
(**a**) Example of SS-J3 samples partially extracted from an SSJ3-D part. Samples remain attached to the sheets in a known configuration until they can be individually labeled. (**b**) Example of a tested SS-J3 sample stored in a bag marked with a unique QR code. The QR code contains information about the printer, build, parent part, and sample location such that it can be uniquely tracked across the digital platform. (**c**) A screenshot of a web-based visual representation of the digital thread for the tensile sample in (**b**). The digital thread provides a record of the operations applied to a sample and its parent part, beginning with printing and ending with tensile testing.

Following heat treatment, the build plate and associated parts were bead-blasted to remove oxide scaling and to provide clean touch-off surfaces to define the part origins for the wire electrical discharge machining (EDM) operation. The parts were separated from the build plate using an AQ750LH (Sodick, Yokohama, Japan) wire EDM and were then fixtured individually for SS-J3 sample extraction. The 3D locations of the SS-J3 samples were predefined in a CAD model, and a tool path was generated using the ESPRIT 2021 (Hexagon, Stockholm, Sweden) computer-aided machining (CAM) software. This CAD model was also sliced using the Magics Image Build Processor for registration with the in situ data (Section 2.2). The parts were sectioned into sheets (Figure 9a), with each individual SS-J3 specimen still attached via a single Table The samples were then manually separated from the surrounding material and placed into individual bags labeled with a quick-response (QR) code (Figure 9b). When scanned, each QR code linked the physical sample to its unique identifier within the digital platform and allowed the retrieval of its digital thread (Figure 9c). This cyberphysical infrastructure substantially aided the robust tracking of the in situ and ex situ data associated with thousands of unique samples.

### 2.8. Tensile Testing Procedure

**Figure 10:**
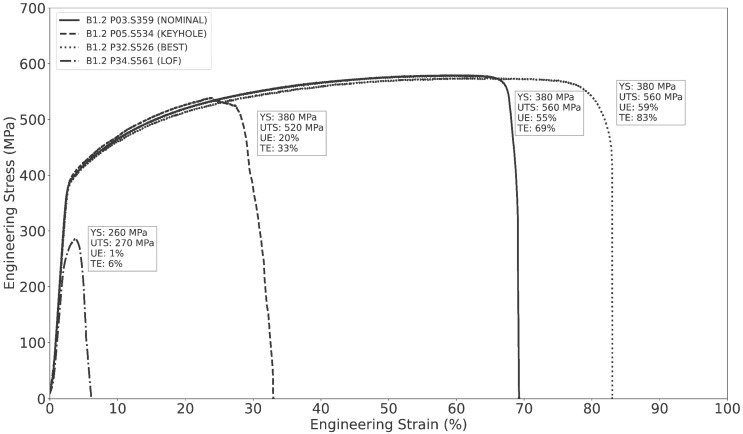
Representative engineering stress–strain curves for four SS-J3 samples extracted from four different SSJ3-D parts printed in B1.2. Each sample was printed with a different parameter set, as indicated in the legend. The calculated values for YS, UTS, UE, and TE are reported next to their corresponding curve. The SS-J3 specimens printed with the best and nominal parameters demonstrate superior static tensile properties compared to the specimens printed with the keyhole and LOF parameters. Strain was approximated by normalizing the crosshead displacement to the nominal gauge length.

Tensile tests were performed using the ASTM E8/E8M [71] procedure with some modifications to facilitate the high volume of testing. Testing was performed across two load frames (TestResources, Shakopee, MN, USA) configured with 500 lbf static-rated load cells calibrated as prescribed by ASTM E4 [72]. The width and thickness of each SS-J3 gauge section were measured using calipers, while the length was assumed to be the nominal value of 5 mm because of the difficulty of accurately measuring this dimension. Samples were installed in a shoulder-loading tensile configuration, preloaded to a nominal load of between 10 N and 50 N, and then continuously loaded under displacement control at a rate of 0.5 mm/min (nominally 10% strain/min). The load and crosshead displacement were recorded at a rate of 10 Hz until the SS-J3 specimen either fractured or the measured load fell below 10 N. Representative engineering stress–strain curves from samples printed in B1.2 are shown in Figure 10.

Tensile properties were algorithmically calculated from the raw load-displacement data. First, the curves were adjusted to account for crosshead displacement measured prior to preloading by shifting the data origin to the initial load measurement and then removing all measurements that were zero load or lower. Then, load and crosshead displacements were converted to engineering stress and strain, respectively, using the initial dimensions of the gauge section. The elastic linear region was identified using datapoints with stress values between 5 and 50 MPa, and the YS was calculated using the canonical 0.2% offset procedure. The UTS was defined as the maximum engineering stress measured during testing, whereas UE was the engineering strain measured from yielding until the point of maximum stress. The TE was defined as the total engineering strain measured from loading until failure of the specimen. Any specimens with UTS values lower than 50 MPa were considered failed tests and were rejected; the lowest non-rejected UTS value was 80 MPa.

### 2.9. Selected Tensile Test Results

Table 5 summarizes the range of tensile property values measured across the five builds, along with reference properties from ASTM A240 [73] and for wrought SS 316L as measured by Byun et al. [64] using similarly sized SS-J2 specimens. Byun et al. [64] also report the effects of a comparable post-build stress-relief heat treatment on the static tensile properties of additively manufactured SS 316L, observing a 46 MPa reduction in YS and no significant differences in UTS, UE, or TE relative to the as-built condition. As desired, the measured tensile properties span a wide range of values across the intentionally varied processing conditions. The minimum YS, UTS, UE, and TE are substantially lower than both the A240 specifications and the wrought properties, whereas the maximum values exceed these baselines. To estimate an appropriate reporting precision for the measured tensile values, the standard deviations were calculated for the non-surface SS-J3 samples extracted from the SSJ3-D part geometry printed with the BEST process parameters in build B1.2. These samples were selected because they were expected to have the lowest true variation in tensile properties, due to uniform thermal conditions and a low flaw density. The measured standard deviations were 16.6 MPa, 15.6 MPa, 1.73%, and 2.92% for YS, UTS, UE, and TE, respectively. Therefore, all tensile values are reported with precisions of 10 MPa, 10 MPa, 1%, and 1%, chosen based on the nearest order of magnitude to the corresponding standard deviation. The complete set of tensile results are available in the Peregrine v2023-11 dataset [44].

**Figure 11:**
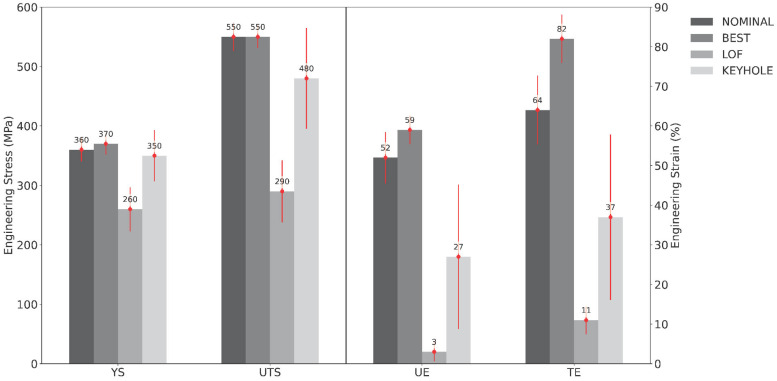
Mean tensile properties measured for all the B1.2 samples extracted from the four SSJ3-D parts, separated by the laser processing parameter set. Each red error bar represents one standard deviation.

Figure 11 shows the mean tensile properties measured for the B1.2 samples extracted from the four SSJ3-D parts, separated by the laser processing parameter set. As expected, the samples printed with the best parameters have the highest tensile properties, followed by the nominal samples. The samples printed with the LOF parameters have drastically lower tensile properties, particularly for UE and TE, which are 56 and 71 percentage points lower than the best values, respectively. Whereas the magnitudes of the keyhole UE and TE values only experience relative reductions of 54% and 55%, respectively, compared to the corresponding BEST values, their standard deviations demonstrate relative increases of 410% and 240%, respectively. This observation suggests that bulk parts produced using the KEYHOLE parameter set may be expected to have substantially increased local variation in their elongation behavior throughout their printed volume as a result of the stochastic formation of individual keyhole pores [59].

**Figure 12:**
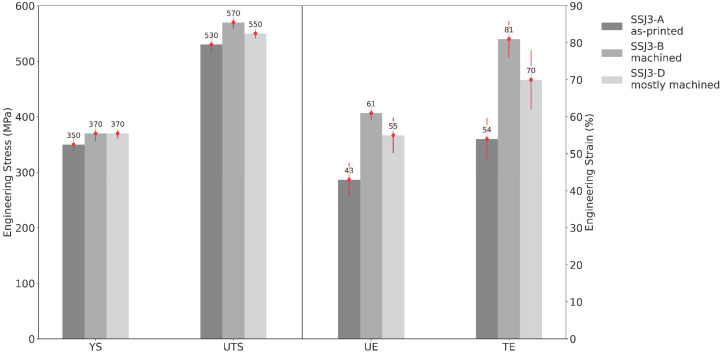
Mean tensile properties measured for all the nominal B1.1 samples separated by the as-printed part geometries. Each red error bar represents one standard deviation.

Figure 12 shows the mean tensile properties measured for the nominal B1.1 samples separated by the as-printed part geometry (i.e., SSJ3-A, SSJ3-B, and SSJ3-D) from which the samples were extracted. The lowest tensile properties were observed for the SSJ3-A thin wall geometry, for which all the extracted SS-J3 samples had two as-printed surfaces. The highest tensile properties were observed for the SSJ3-B thin wall geometry, for which all the extracted samples had only machined surfaces. Additionally, the SSJ3-B thin wall is expected to have a different thermal history than the SSJ3-D blocks, which may result in beneficial microstructural differences [66]. However, this has not been specifically investigated for these samples.

**Figure 13:**
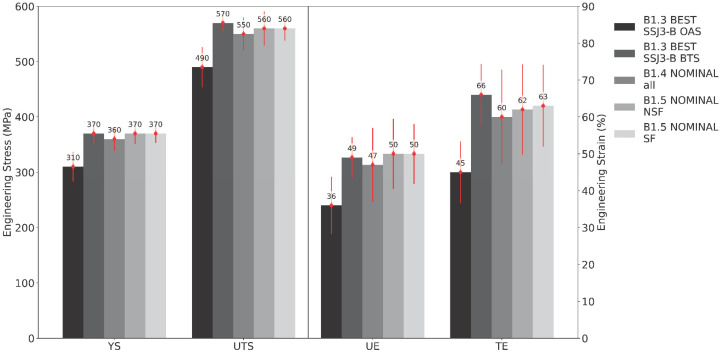
Mean tensile properties measured from several selected subsets of samples from builds B1.3, B1.4, and B1.5. Each red error bar represents one standard deviation.

Figure 13 shows the mean tensile properties measured across selected subsets from builds B1.3, B1.4, and B1.5. Considering B1.3, the best overhang-adjacent surface (OAS) samples extracted from the SSJ3-B parts have substantially lower tensile properties compared to the corresponding best bulk and top surface (BTS) samples. This indicates that printed material immediately adjacent to an overhanging design feature can be expected to have noticeably reduced tensile properties. Conversely, the nominal subset from B1.4 suggests that the increased amount of soot does not substantially impact the static tensile properties relative to the nominal data from B1.2. Similarly, comparing the nominal no-short-feed (NSF) and nominal short-feed (SF) subsets from B1.5 indicates that the intentional powder short feeds (caused by the reduction in dosing factor) had no significant impact on any of the measured tensile properties. Further analysis of this large quantity of tensile data is beyond the scope of this work.

### 2.10. Construction of the Super-Voxels

**Figure 14:**
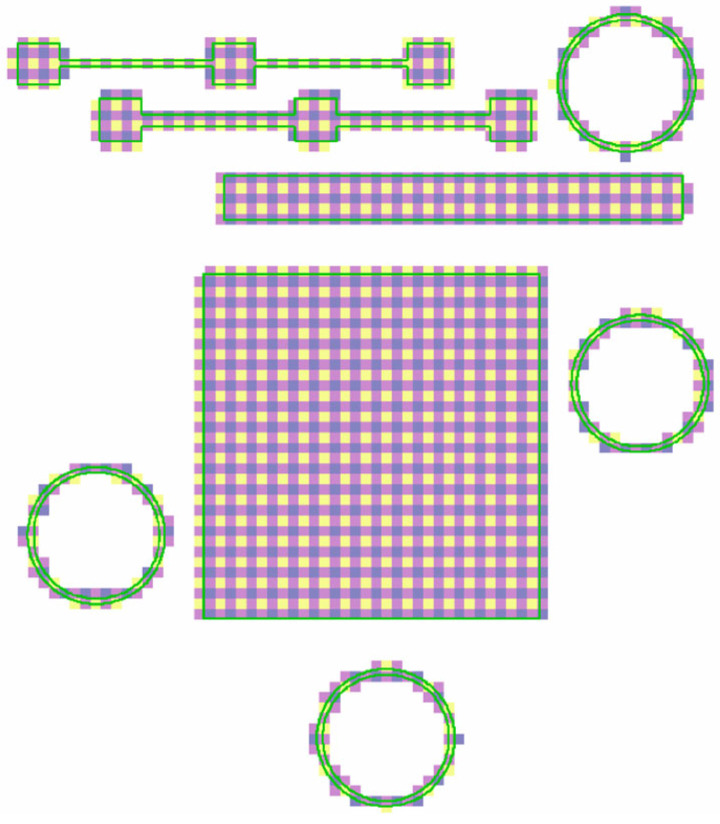
Intersection of the fixed super-voxel grid with a set of CAD geometries (green lines). Only super-voxels that overlap the CAD geometry are shown. Each super-voxel is 1 mm on a side and is colored so that its boundaries with neighboring super-voxels are visible.

To allow an ML model (N6) to learn an accurate transfer function between the in situ data and the tensile properties measured during testing, the in situ sensor data and part geometry information were encoded into engineered feature vectors corresponding to discrete volumes designated as super-voxels (N5). The encoding was designed to contain the information required for a generalizable ML model to make property predictions at the super-voxel size scale. To this end, the printer’s build volume was demarcated into a fixed grid of rectangular prisms, with each super-voxel measuring 1.0 × 1.0 mm in the *x*-*y* plane and 3.5 mm along the *z*-axis. Several factors, including empirical measures of model performance, were considered when determining an appropriate super-voxel size. First, the super-voxels were sized to approximately match the volume of the SS-J3 gauge sections, which were, in turn, sized based on the criteria enumerated in Section 2.5 and Appendix A. Of secondary importance, computational memory restrictions placed a lower bound on the super-voxel volume. Figure 14 shows a portion of a print layer divided into a super-voxel grid, with super-voxels extending 70 layers in the vertical print direction.

**Figure 15:**
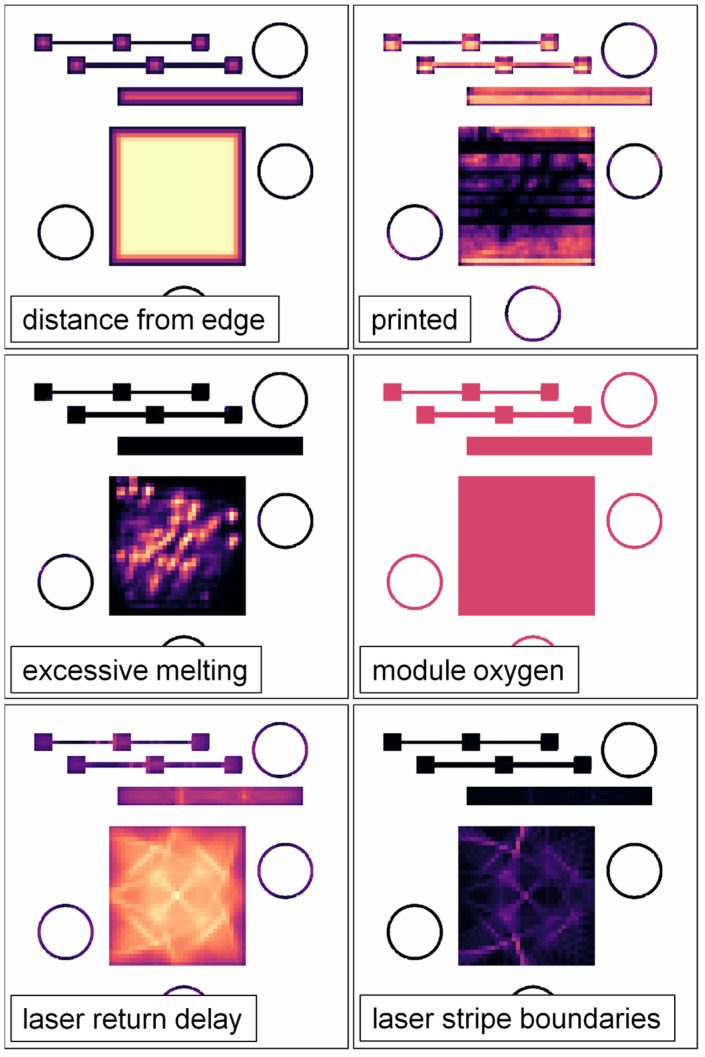
Normalized visualizations of six super-voxel features with brighter and darker regions, respectively, indicating larger and smaller values. These super-voxels encode data from the keyhole parts for layers 639 (31.95 mm) through 709 (35.45 mm) of B1.2. For ease of interpretation, super-voxels not fully contained within the CAD geometry have been cropped. Note that the feature encoding the distance from the part edge saturates in the center of the SSJ3-D part. Also note that the module oxygen value is uniform for all super-voxels fully contained within the same layer range.

Each feature vector was composed of 21 engineered features calculated based on the CAD geometries, pixel-wise DSCNN segmentations of the post-melt and post-spreading visible-light layer images, sensor data recorded in the printer log file, and laser scan path data. Each feature is enumerated, described, and justified in Appendix D. Figure 15 shows six example feature maps for a sub-region of B1.2. The planar resolution of the geometry information, DSCNN results, and laser scan path data was 130 µm (Section 2.2), which is substantially smaller than the super-voxel size. Therefore, these pixel-wise values were first averaged in the *x*-*y* plane to produce a single value per feature, per super-voxel, per layer. To mitigate computational edge effects, only pixels contained within the CAD geometry were considered in the planar averaging. Similarly, the vertical resolution of these features and the log file data was equal to the 50 µm layer thickness. Therefore, averaging in the vertical direction was accomplished with a sliding window filter with a stride equal to the height of the super-voxels. For super-voxels intersecting the edges of the printed part geometries, the averages are weighted proportionally to the number of pixels contained within the intersection of the super-voxel and the CAD geometry at each layer.

As discussed in more detail in the following section, the training data for the tensile property prediction model consisted of the feature vectors for valid super-voxels intersecting the SS-J3 gauge sections. Super-voxels were considered invalid if any in situ data were missing or if less than 10% of their area overlapped with the gauge section of a given SS-J3 sample. To improve training stability, the raw feature values are zero-center normalized on [−1,1] based on the minimum and maximum values observed across the combined training and validation dataset. Generally, the potential for an ML model to learn meaningful discrimination between material volumes increases as the number of in situ sensor modalities and engineered features increases. However, the total number of features must be balanced with the challenges of performing enough experiments (Section 2.6) to capture the expected variation across each feature axis.

### 2.11. Architecture and Training of the Property Prediction Model

The voxelized property prediction model (VPPM) implements a perceptron [74] ML algorithm and was used to predict the local tensile properties at N6 based on the engineered feature vectors described in Section 2.10. The ground truth training targets are the zero-center normalized tensile measurements reported in Section 2.9. Perceptrons are shallow neural networks [16] that have been successfully applied to a wide range of regression problem sets. The perceptron is not necessarily the optimal ML model for this stage of the relay, but it serves as a viable proof-of-concept because this work primarily focuses on framework development, data collection, and feature engineering, leaving model optimization for future publications. A separate VPPM was trained to predict each of the four tensile properties: YS, UTS, UE, and TE. The perceptron architecture is reported in Table 6. Along with the training procedure, the perceptron architecture is identical for each of the VPPMs. The fully connected and dropout neural network layers used by the VPPM are described in Krizhevsky et al. [75].

The VPPMs were trained using backpropagation [76] and the Adam optimizer [77] with a loss function that minimized the *L*_1_ (i.e., absolute) error between the predicted values and the ground truths. The kernel weights of both fully connected layers were initialized from zero-centered normal distributions with a standard deviation of 0.1. Each VPPM was trained using a mini-batch size of 1000 super-voxels and a learning rate of $1\times 10^{-8}$, with tests indicating that the model performance was insensitive to these hyperparameters. The first and second exponential decay rates of the Adam optimizer were set at 0.9 and 0.999, respectively, and the epsilon value was fixed at $1\times 10^{-4}$.

The 6299 SS-J3 tensile samples available for training corresponded to 29,680 unique super-voxel feature vectors, with an average of 4.7 super-voxels per SS-J3 gauge section. The mini-batch size was 1000 super-voxels, with 20% of the data used for validation during each repetition of a 5-fold cross-validation procedure [78]. Cross-validation was performed to reduce the sensitivity of the model performance to the randomly determined split between training and validation datasets. For each fold repetition, training proceeded until the validation error plateaued. Bifurcation of the data into the training and validation sets was performed sample-wise so that all super-voxels associated with the same ground truth were assigned to the same set. Note the implication that the same ground truth target, i.e., measured SS-J3 tensile property value, is associated with multiple super-voxels, the exact number of which depends on the intersection of the fixed super-voxel grid with the sample geometry in 3D space. This condition caps the maximum possible model performance and, in extremis, could cause instabilities in the training process because each ground truth cannot be correlated to a unique feature vector. Therefore, minimizing the number of super-voxels contained by each gauge section was a key computational consideration for setting the appropriate super-voxel size. Ultimately, this limitation is dependent upon the localizability of the ex situ testing data and is further considered in the Discussion section. Although VPPM validation accuracy might be improved by calculating each feature vector based on the totality of the SS-J3 gauge volume instead of for super-voxels on a fixed grid, such an approach would not be representative of model test conditions (i.e., predicting local properties for a printed component of arbitrary geometry) because the prediction volumes would be differently sized, and certain edge effects (e.g., varying overlap between the super-voxels and the part geometry) would not be accounted for in the training or validation sets.

At model test time, each VPPM applied its learned weights and biases to each feature vector, predicting a single value for each super-voxel that was then denormalized and converted to real YS, UTS, UE, or TE units. For part qualification, it is valuable to identify predictions that may be extrapolated instead of interpolated to ensure that human decision-makers are involved whenever conditions exceed the scope of the models’ training. This area of study is commonly referred to as *out-of-distribution detection* [79] and is a nontrivial determination in high-dimensional space. For this work, a super-voxel was considered out-of-distribution if the value of any one of the individual features was below the minimum or above the maximum values observed for that feature within the training set. Although this bounding heuristic is a necessary check, it is not strictly sufficient. A more sufficient approach might consist of a clustering analysis [80] of the training set paired with an empirically determined maximum acceptable distance between a new feature vector and the observed clusters. However, advanced out-of-distribution detection methods are reserved for future work. Computationally, any predictions for out-of-distribution super-voxels were converted to not-a-number (NaN) values and were reported separately.

## 3. Results

### 3.1. Validation Performance

The primary performance metrics used to evaluate the VPPMs, and by extension, the entire property prediction pipeline, were based on the root mean square (RMS) validation errors averaged over five training folds. Because each gauge section contains several super-voxels, multiple unique predictions were made for sub-volumes within each sample. These overlapping predictions were collapsed to a single value by calculating the minimum of these predictions. Taking the minimum value provides a conservative estimate of the local material properties, which best informs the part qualification process. Averaged over the five folds, only 0.05% of super-voxels were considered out of distribution, indicating that the training sets successfully captured the process variability observed in the validation set.

Table 7 reports the average RMS error and standard deviations for each VPPM trained using the full set of 21 features. In relative terms, the RMS errors ranged between 7.1% and 13.2% of their corresponding observed property ranges. It is worthwhile to compare the VPPM errors to the RMS error produced by naïvely predicting the average of the ground truth property values observed across the entire dataset. Importantly, this comparison demonstrates error reductions between 30% and 48% over the naïve approach, which assumes average tensile properties throughout an entire L-PBF part, with the largest improvement reported for the UTS predictions. Next, the total RMS error can be considered as the summation of the intrinsic measurement error (i.e., aleatoric uncertainty) ${RMSE}_{I}$ of the tensile test and the model error ${RMSE}_{M-VPPM}$ as shown in Equation (4), where the intrinsic measurement error cannot be predicted by any model and is estimated by calculating the standard deviation (equivalent to an RMS error in this situation) for the subset of BEST SS-J3 samples extracted from the non-edge regions of the SSJ3-D part produced in B1.2 (see Section 2.9 for justification). This intrinsic error is also a component of the naïve RMS error (${RMSE}_{naïve}$) as shown in Equation (5) and can, therefore, be separated from both the VPPM and the naïve predictions. After separating the intrinsic error, the relative reductions in the errors improve to 57%, 61%, 49%, and 46% for YS, UTS, UE, and TE, respectively. Because this final metric considers both the distribution of the data due to the process variation across the entire sample set and the spread associated with intrinsic variations in the tensile testing procedure, the authors propose the use of this or similar metrics when comparing these VPPM prediction results with those reported for similar works in the future. Although this is beyond the scope of this work, a more rigorous analysis of the summation of the error terms [81] may further improve the utility of such performance metrics.

(4) $${RMSE}_{VPPM}={RMSE}_{I}+{RMSE}_{M-VPPM}$$

(5) $${RMSE}_{naïve}={RMSE}_{I}+{RMSE}_{M-naïve}$$

To infer the relative importance of the different features, additional VPPMs were trained using three subsets of the available features: the first set was composed of only the CAD (Table A2) and scan path information (Table A5), the second set was composed of only data from the printer log file (Table A4), and the third set was composed of only the DSCNN segmentation results (Table A3). Comparing the results presented in Table 8 to those in Table 7, it is apparent that the VPPMs with access to only the CAD information, scan path information, and printer log file data have predictive powers comparable to the naïve approach (Table 7). However, including these features in the full-featured VPPM results in a minor reduction in the RMS errors relative to the VPPMs trained with the DSCNN segmentations alone. To estimate the sensitivity of the model performance to the size of the training set, an ablation study was performed in which the size of the training set was artificially reduced to 20% of the available data. As shown in Table 8, the RMS errors remain essentially unchanged, suggesting that significantly fewer tensile tests could be performed without negatively affecting the predictive capabilities. Reducing the size of the training set below 20% was not explored in this work and will require careful consideration to ensure that a representative sampling of the expected process variations is maintained. Additional validation metrics may also be required to properly measure any increases in the prediction error for relatively rare events and process conditions.

**Figure 16:**
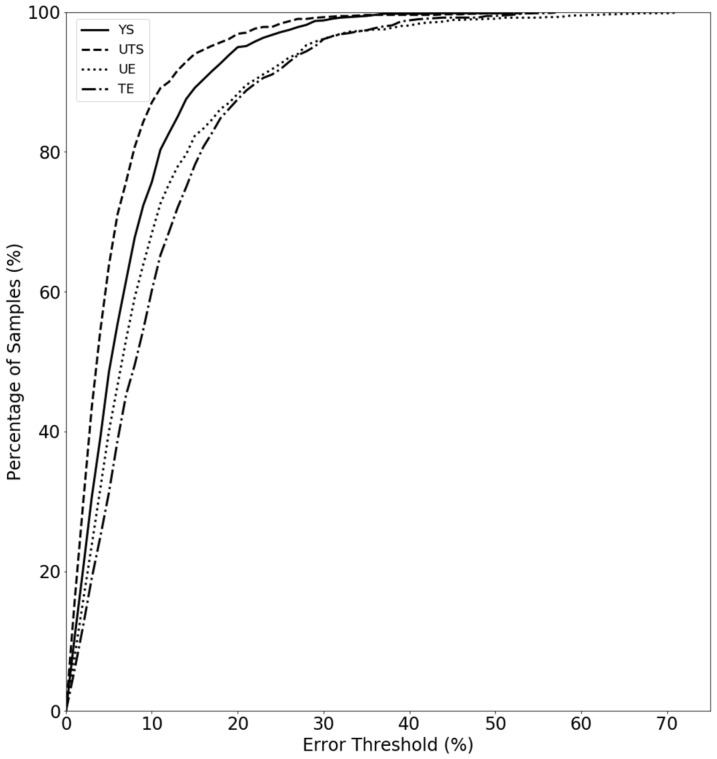
Validation ROC-like curves for each of the four VPPMs, as indicated in the legend. The *y*-axis reports the percentage of validation samples with RMS errors less than the error threshold given on the *x*-axis. The RMS errors are reported as a percentage of the observed range of the corresponding tensile value within the validation set.

The following results use VPPMs that were trained using the full feature set from the same representative fold iteration. Figure 16 plots curves similar in function to receiver operating characteristics (ROC) curves [82] for each of the four VPPMs. The *y*-axis reports the percentage of validation samples with RMS errors less than the error threshold, given as a percentage of the observed validation range of the corresponding tensile value, on the *x*-axis. For example, 81% of the validation samples have UTS RMS errors less than 8.0% of the observed validation range of 470 MPa. As suggested by the metrics above, the UTS VPPM demonstrates the strongest predictive ability, whereas the UE and TE VPPMs demonstrate the weakest performance. Interestingly, although the UE VPPM slightly outperforms the TE VPPM at lower error thresholds, its relatively longer tail at higher error thresholds suggests that a small number of samples have measured UE values that are particularly difficult for the trained VPPM to predict accurately.

**Figure 17:**
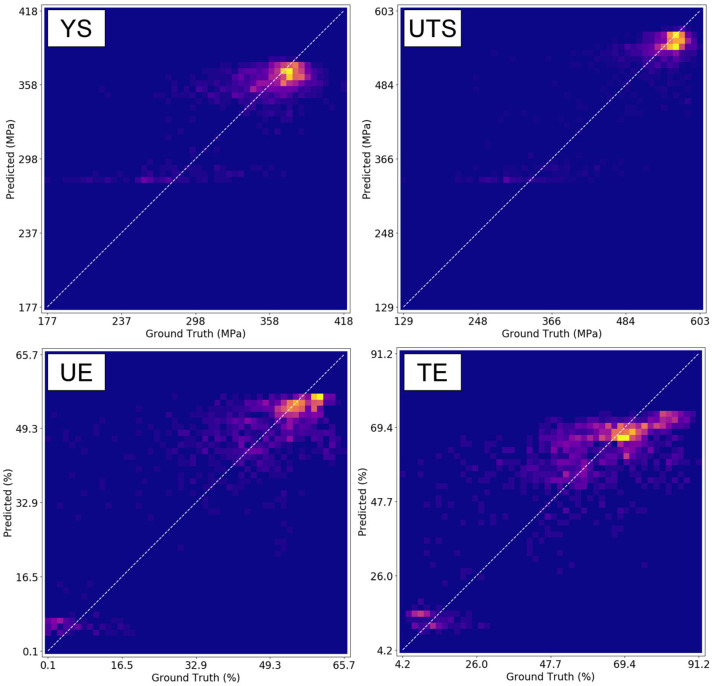
Normalized correlation plots showing VPPM predictions for YS, UTS, UE, and TE vs. the corresponding measured ground truth values for a selected validation fold. The color map indicates the number of SS-J3 samples present in each bin, with more brightly colored bins containing more samples and the darkest bins containing no samples. If each VPPM performed perfectly, then all the datapoints would lie on the diagonal dashed line with a slope of unity.

Figure 17 shows correlation plots for each of the four VPPMs for a selected validation fold. In these plots, the *x*-axis reports the ground truth tensile measurement, and the *y*-axis reports the predicted tensile property value. This representation of the validation accuracy can be considered a 2D histogram, with the colormap representing the number of SS-J3 samples present in each bin. If all the VPPM predictions were correct, then only the bins along the diagonal line (with a slope of unity) would be brightly colored. Note that the ground truth YS and UTS measurements are primarily bimodal, whereas the UE and TE measurements exhibit a substantially more uniform distribution of values spread across the observed range. At the time of writing, the cause of this difference in distribution behavior has not been determined.

**Figure 18:**
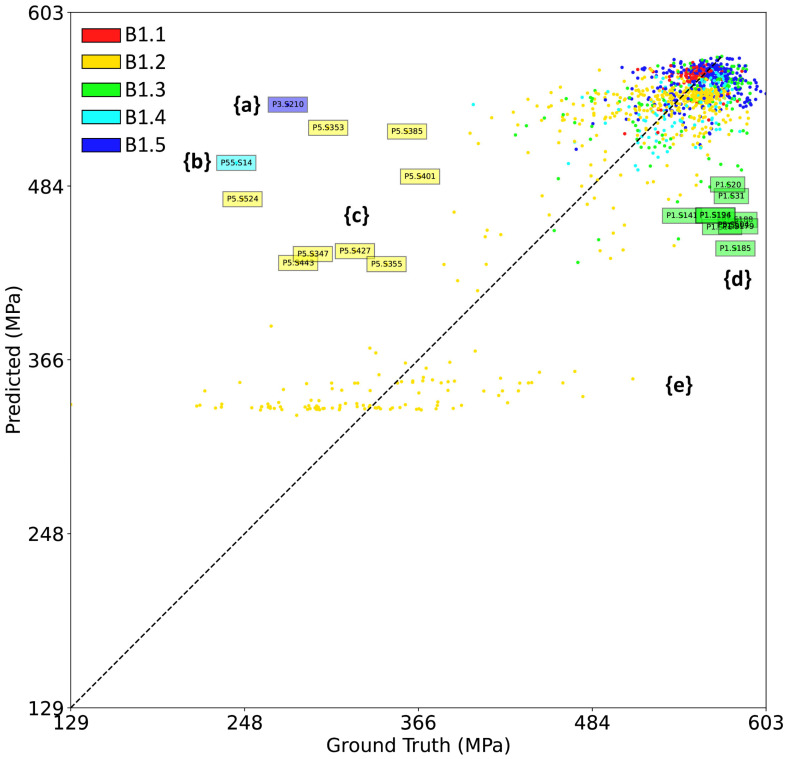
Scatter plot showing the UTS VPPM predictions vs. the corresponding measured ground truth values for a selected validation fold. Each datapoint represents a single SS-J3 sample tracked by a unique identifier using the digital platform. The datapoints are colored by the source build as identified in the legend. If the UTS VPPM performed perfectly, then all the datapoints would lie on the diagonal dashed line with a slope of unity. Selected subsets of the outlying datapoints are marked by letters enclosed by curly brackets and are discussed in the text.

To focus on the outlying predictions, Figure 18 shows a scatter plot of predicted UTS versus the ground truth values for a selected validation fold. Each datapoint represents a single SS-J3 gauge section, and some are labeled with the automatically generated unique identifier matching the sample’s physical QR code and tracked by the digital platform. If all the UTS VPPM predictions were correct, then all the datapoints would lie on the diagonal line with a slope of unity.

**Figure 19:**
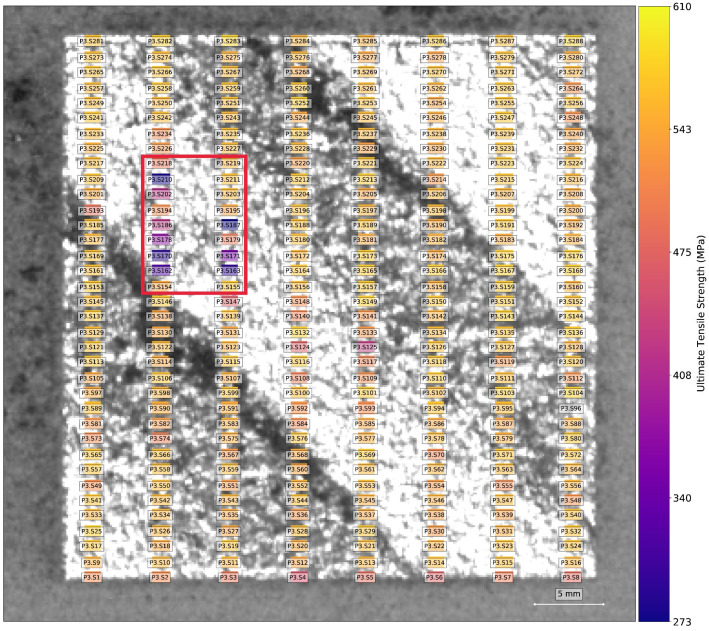
Measured UTS values for 288 uniquely identified SS-J3 tensile specimens extracted from an SSJ3-D part printed in B1.5, spatially registered to the corresponding CAD coordinate system. For reference, test results are overlaid on top of the visible-light post-melt image captured for the layer located in the center of their gauge sections. Within the color space, brighter samples represent a higher measured UTS, and darker samples represent a lower measured UTS. Note the cluster of relatively low UTS measurements highlighted by the red bounding box.

Outlier set {a} represents a single tensile sample, designated as P3.S210, from build B1.5 that is predicted to have a UTS of 540 MPa but has a measured UTS of 280 MPa. Sample P3.S210 was extracted from the bulk region of an SSJ3-D part printed with nominal process parameters. As shown in Figure 19, P3.S210 is surrounded by several other samples with UTS values that are substantially lower than the rest of the printed part. Samples in this group were not tested sequentially, so it is likely that this outlier represents real variation in the printed material (as opposed to a tensile testing artifact) not properly modeled by the VPPM. Future investigation of the fracture surfaces may indicate the root cause of this behavior.

Outlier set {b} also represents a single tensile sample (P55.S14 from a nominal SSJ3-B part printed in B1.4) that is predicted to have a UTS of 500 MPa but has a measured UTS of 240 MPa. In contrast to set {a}, the samples surrounding P55.S14 were all measured to have much higher UTS values, including sample P55.S17, which is located at the same distance from its closest soot generator and is expected to have a similar thermal history. Additional investigation (e.g., microstructural characterization), which is beyond the scope of this paper, would be necessary to determine if this measurement represents a true variation in the material properties or if it is an artifact of the tensile testing procedure.

Outlier set {c} represents a group of samples extracted from an SSJ3-D part (designated P5) produced using keyhole parameters in B1.2, which were measured to have UTS values ranging from approximately 250 to 450 MPa. Although the VPPM successfully predicts some of this variation, it is not fully captured by the model. The authors hypothesize that the relatively large vertical dimension of the SS-J3 gauge sections is detrimental to the predictive performance of the VPPMs for material with significant populations of keyhole porosity because it is difficult to correlate the volumetric tensile measurements with individual in situ sensor indications of keyholing.

Next, outlier set {d} represents a group of samples extracted from an SSJ3-C part (designated P1) printed using best parameters in B1.3, which were measured to have UTS values of approximately 570 MPa but were predicted to have a UTS as low as 440 MPa. A manual review of the sensor data for P1 revealed that significantly more soot was generated in the vicinity of this part than is typical for parts produced using the best parameters or for the other parts printed in B1.3. This soot was correctly segmented by the DSCNN, and the increased soot levels were encoded into the corresponding super-voxel feature vectors. Unlike other cases within this dataset (e.g., the LOF parts), these increased soot levels did not correlate with a lower measured UTS, resulting in the lower-than-correct VPPM predictions.

Finally, outlier set {e} encompasses samples extracted from the LOF parts produced in B1.2. These samples exhibited extremely low UTS values (Figure 11) because of the significant levels of lack-of-fusion porosity present within the parts. Significant variation in UTS is also observed, likely the result of the variable number of pores that intersect with a given SS-J3 gauge section. After a manual review of the sensor data, the authors consider it unlikely that this variation could be more accurately modeled, given the available in situ sensor data. Fortunately, the VPPM still correctly recognizes that these samples have much lower strengths than nominal L-PBF SS 316L material, which would be a sufficient threshold for many part qualification scenarios.

### 3.2. Testing Performance

Extraction of the CAD geometries, spatial registration of the visible-light camera data, and anomaly segmentation by the DSCNN were performed in real-time on a network-attached server during the printing operation, and they collectively required several seconds of processing time per print layer, as discussed in Section 2.4. The time required to calculate the feature vectors for all the super-voxels within a build depended upon the volume of printed material and ranged from 12 to 19 h for the five builds reported in this work and was performed post-build. Once the feature vectors were generated, the prediction of the local tensile properties using the trained VPPMs was trivial, requiring less than a minute for each build. Given the early-stop criteria, VPPM training required less than five minutes for each fold. Therefore, the most computationally expensive portion of this AIR was the generation of the super-voxel feature vectors. Currently, super-voxel generation is dominated by the time required to retrieve the scan path information from the TDMS files and spatially map the corresponding temporal data into the common spatial coordinate system [51]. The authors expect that additional computational optimizations can be applied to reduce this time burden significantly.

**Figure 20:**
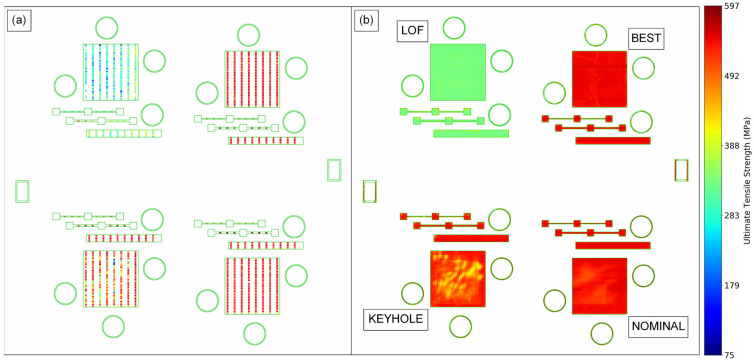
(**a**) Measured UTS values from the SS-J3 samples located between layer 639 (31.95 mm) and layer 709 (35.45 mm) of B1.2. (**b**) VPPM-predicted local UTS values for the corresponding layers as a color map, with white super-voxels indicating regions beyond the part geometries or detected instances of out-of-distribution super-voxels. The nominal CAD geometry is indicated by the green outlines, and both images show the full 245 × 245 mm print area.

Figure 20 shows the local UTS predictions for a set of layers from B1.2 alongside the UTS values measured from the SS-J3 samples extracted from the same vertical region of that build. Most noticeably, the models correctly predict the low strength of the LOF parts in the back-left quadrant, although the exact values are nearly a uniform average of the observed variation for these parts, as discussed in Section 3.1. Importantly, the property variation measured for the keyhole SSJ3-D part in the front-left quadrant is also captured, with structures appearing qualitatively analogous to the DSCNN segmentations of excessive melting shown in Figure 15. Furthermore, despite the measured UTS differences being relatively small, the models also correctly predicted slightly lower strengths for the thin-walled SSJ3-A parts, as well as other parts with similarly thin cross-sections. Interestingly, within the nominal SSJ3-D part in the back-right quadrant, regions of slightly lower strength were predicted that qualitatively overlap with several of the structures observed in the scan path feature maps (Figure 15); additional investigation would be required to determine the veracity of this predicted shift in UTS distributions.

**Figure 21:**
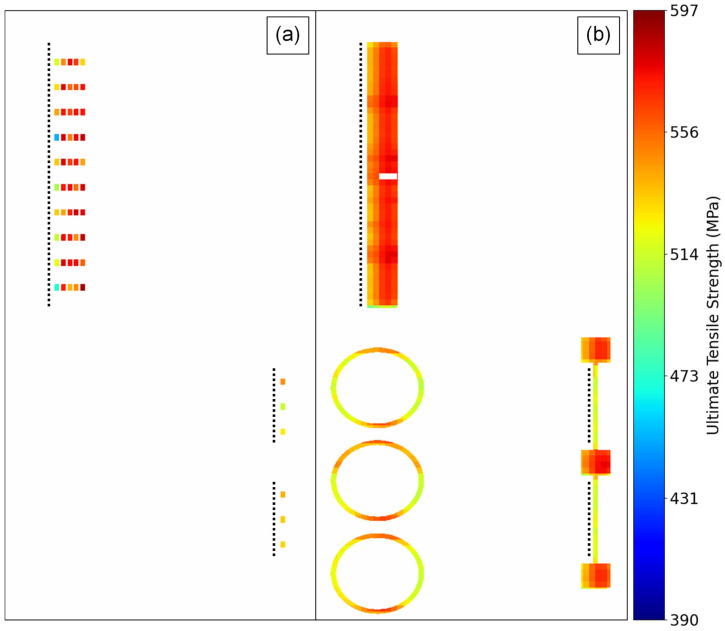
(**a**) Measured UTS values from the SS-J3 samples located within a region of interest and between layer 1136 (56.80 mm) and layer 1206 (60.30 mm) of B1.3. The region of interest lies within the back-right quadrant of the powder bed, cropped so that the right side of the image is 122.5 mm in length. (**b**) VPPM-predicted local UTS values for the corresponding layers as a color map, with white super-voxels indicating regions beyond the part geometries or detected instances of out-of-distribution super-voxels. Overhanging surfaces of interest are indicated by dashed vertical lines.

As a final example, Figure 21 shows the local UTS predictions within a region of interest for a set of layers from B1.3 alongside the UTS values measured from the corresponding SS-J3 samples. The models correctly predict that the UTS was, on average, substantially reduced in the thin-walled SSJ3-A parts and for super-voxels immediately adjacent to the overhanging surface of the thicker SSJ3-C parts (Figure 13). Of course, with access to entire volumes of localized tensile property predictions, a wealth of additional visualizations and analyses are possible. However, the authors reserve a more detailed exploration of volumetric property predictions for future work.

## 4. Discussion

In this study, the authors demonstrated several key components of a novel L-PBF part qualification framework. The results of the tensile tests reported in Section 2.9 make apparent the interdependencies between local part geometry, melting parameters, and process conditions, which challenge efforts to create traditional manufacturing design rules for L-PBF AM. Fortunately, as shown in Section 3.1 and Section 3.2, the relay of machine-learned models (i.e., the AIR) achieved direct localized prediction of tensile properties for L-PBF printed parts based on data collected in situ during the printing process. The presented framework is designed to be geometry- and process-parameter-agnostic and, therefore, substantially generalizable. Importantly, each step in the proposed property prediction pipeline is modular, allowing the iterative improvement of each component to address specific limitations. During this study, the authors identified four areas that should be addressed before similar AIR-based pipelines are applied to instance-wise part qualification.

First, the resolution of the visible-light camera is currently limited, with a resolving power of only 280 µm and many features that might indicate sub-surface pores or other flaws are not currently observable. Other work has demonstrated the successful use of substantially higher resolution visible-light cameras for this purpose, such as the analysis by Snow et al. [83], which used a 36.3 MP sensor to image a similarly sized powder bed. Similarly, multiple lighting conditions can be used to cast shadows across the powder bed and increase the contrast of topological features associated with various processing anomalies [83]. The incorporation of temporally integrated near-infrared (NIR) camera data, as explored by Schwerz [23], may allow the DSCNN to segment additional anomaly classes, such as individual spatter particles interfering with the melt tracks [84]. Indeed, the performance of the current models is impressive, given that a low-cost, OEM-standard sensor suite was used. The authors expect that significant performance improvements can be achieved with improved sensor modalities.

Second, precise localization of the tensile test results remains a challenge. Even using the subsize SS-J3 specimens, the measured tensile properties apply to a relatively large volume of printed material compared to the size of stochastic flaws such as keyhole porosity. A potential solution is to use digital image correlation (DIC) [85] to further localize the failure location within the specimen’s gauge section. Additionally, a subset of the SS-J3 specimens could be rotated during printing so that their long axis is perpendicular to the vertical build direction. This approach could enable better correlation between tensile measurements and certain laser scan path features and vertically isolated flaws such as powder short feeds. Further shrinking the gauge section is also possible but will have to be considered carefully to avoid introducing additional mechanical testing artifacts. Relatedly, intrinsic tensile measurement errors could be reduced by using DIC to observe the true strain (as opposed to using crosshead displacement as a proxy), measuring the initial gauge section dimensions using an optical silhouette or laser profilometer technique, and using a robotic arm to insert the specimens into the tensile load frames repeatably.

Third, based on the results of this work, each of the DL, ML, and feature encoding steps can now be improved. Currently, all the DSCNN annotations are generated manually, but some flaw indications observable in the NIR [23] sensor modality could be annotated automatically using XCT as the ground truth [83]. Furthermore, in this work, local thermal histories are only represented in the super-voxel feature vectors via proxy, but the use of contemporary analytical thermal models could instead directly encode the thermal histories, as shown by Donegan et al. [46] and Stump and Plotkowski [86]. Similarly, the current feature vectors do not explicitly discriminate between super-voxels with machined vs. as-printed surfaces. This limitation was most apparent in the predictions for the SSJ3-A and SSJ3-B part types, both of which had similar wall thicknesses but different surface conditions. Indeed, this situation demonstrates the need for the digital thread to include information beyond just the printing operation itself (i.e., post-processing steps). Finally, the VPPM perceptrons were trained using a canonical *L*_1_ loss function, which may bias the model to learn average responses at the expense of predicting rare events. Applying a loss weighting scheme, such as that described for the DSCNN in Section 2.3, or implementing a different ML model type altogether, may improve the ability to model the effects of rare processing conditions. Notably, the existence of several outlying predictions suggests that the VPPM is not overfitting to specific fluctuations in the data. For example, outlier {a} is representative of several co-located SS-J3 specimens (Figure 19) with unusually low measured UTS values but in situ sensor signatures highly similar those of specimens with much higher UTS values. The VPPM incorrectly predicts the UTS for all nine of these outlying specimens, including those in the training set. If the VPPM were overfitting, it is expected that it would instead correctly predict outliers in its training set, despite the lack of physically significant variation in their corresponding input features. Therefore, the depth of this neural network could potentially be increased to improve performance while still producing a generalizable model.

Lastly, if any data-driven L-PBF qualification framework is to be adopted by an industrial user base, it cannot be prohibitively expensive to implement at scale. Therefore, the generalizability of the proposed framework to different L-PBF printers, feedstock materials, and material properties is a critical consideration. For discussion, the authors have grouped potential model transfer situations into three categories of increasing difficulty. In the first category, the authors hypothesize that a relay of pre-trained models could be directly transferred to other similar L-PBF printers using the same alloy, assuming that the in situ sensor suite is held constant. In the next category, while the proposed data analysis framework could be applied to a different alloy or material property, this approach will certainly require replication of a significant testing campaign and a retraining of the machine-learned models. Fortunately, the ablation results presented in Section 3.1 suggest that the number of mechanical tests could be reduced by at least 80% without significantly degrading the predictive capabilities. However, additional research is necessary to determine the minimum number of specimens required to fully represent the possible process conditions within the training set, and alternate measures of VPPM performance may be needed to ensure that the error rate for predicting rare but safety-critical conditions is accurately quantified. It is also apparent that some material properties (e.g., fatigue life) are substantially more expensive to measure at scale than static tensile properties, so the bypass between N4 and N7 (Figure 1) utilizing analytical modeling may be the more industrially scalable option for such properties. Alternatively, recent federated learning strategies such as those proposed by Mehta and Shao [30] offer a potential solution to this problem whereby expensive specimen characterization could be performed across multiple locations but used to jointly train common models without compromising the intellectual property of any of the participants. In the final category, it must be recognized that the difficulty of observing certain anomalies in situ will vary across alloy systems, just as the localizability of the ex situ measurements will depend on the material property of interest. In these situations, alternate data-driven qualification frameworks should be explored.

## 5. Conclusions

In this work, the authors implemented a relay of machine-learned models to further digital twin-informed, instance-wise qualification of parts printed using L-PBF processes. The presented modular architecture was designed to be flexible, providing a conceptual framework for future AM part qualification efforts. To support these efforts, the authors printed and tested over six thousand tensile specimens, each of which was uniquely tracked and spatially registered to the in situ sensor data using a cyberphysical digital platform. The co-registered in situ data and tensile test results are approximately 230 GB in size and are available in the Peregrine v2023-11 dataset [44]. Layer-wise visible-light images of the powder bed were segmented using a DSCNN, the results of which were combined with other in situ data and then encoded into human-engineered feature vectors using a combination of image segmentation neural networks and classical signal processing and computer vision algorithms. Localized tensile properties were then predicted at the super-voxel scale by training a perceptron neural network with the ground truth tensile measurements.

The viability of this framework was evaluated with multiple performance metrics, demonstrating significant error reductions relative to traditional, naïve estimates of several tensile properties, with the best performance improvements observed for UTS. An ablation study indicated that the in situ layer-wise visible-light powder bed images contained the majority of the information used by the trained models to predict the tensile properties, while the log file data, laser scan paths, and CAD information enabled only moderate improvements. Several sets of outliers were explored in detail to better understand the generalizability of the learned models and to motivate future work. Finally, tensile properties were predicted for the entire volumes of all five builds, enabling the qualitative assessment of the framework’s ability to capture experimentally observed trends, such as the effects of process parameters, wall thickness, and overhanging surfaces.

Three primary areas have been identified for future work. First, the authors will make iterative improvements, such as increasing the visible-light imaging sensor resolution and incorporating a temporally integrated NIR imaging system as an additional in situ sensor modality. Additionally, the use of a robotic tensile testing system and DIC will enable increased sample throughput and will provide more accurate measurements of the specimen gauge sections to reduce the overall intrinsic tensile measurement error. Changes to the specimen design and build layout will also improve the localizability of the ground truth data and will prioritize building smaller sets of samples distributed across a wider range of builds. Next, the authors will seek to quantify the generalizability of the framework by applying it to other alloys, L-PBF printers, sensor modalities, and material properties. Because this set of work will span efforts across multiple national laboratories, it may also explore techniques such as federated learning, which will be critical for the industry to adopt any artificial intelligence-based qualification frameworks relying on extensive training datasets. Finally, software tools and experiments must be developed to demonstrate the ability of the proposed approach to estimate the performance of a given instance of a part within a specific application context. Ultimately, the authors believe that this work demonstrates one of the first viable approaches for direct, localized material property prediction based on in situ sensor data collected during an L-PBF printing process.

## Figures and Tables

**Table 1:** The TP and FP validation performance metrics for the specific DSCNN model used. The training and validation dataset splits are 90% and 10%, respectively. FP rates are typically higher for spatially small classes owing to the chosen balance between the class-wise and interfacial boundary loss weighting schemes.

Class	TP (%)	FP (%)
Powder	97.3	0.8
Printed	98.6	5.1
Recoater streaking	87.4	62.8
Edge swelling	95.1	34.2
Debris	95.9	26.2
Super-elevation	98.5	3.0
Soot	85.8	15.5
Excessive melting	94.8	72.2

**Table 2:** Chemical composition of the SS 316L powder lot as reported by Praxair. Values are given in weight percent.

C	Co	Cr	Cu	Fe	Mn	Mo	N	Ni	O	P	S	Si	Other
<0.005	0.08	17.01	0.00	Bal	1.29	2.48	0.01	12.67	0.03	<0.005	0.005	0.59	<0.1

**Table 3:** Laser raster parameter sets used across the tensile sample builds, as provided by Concept Laser or determined through internal testing.

Parameter Set	Laser Beam Power (W)	Laser Beam Speed (mm/s)	Hatch Spacing (µm)	Nominal Laser Spot Size (µm)	Stripe Width (mm)	Scan Rotation (Degrees/Layer)
Nominal	370	1350	90	130	10	67
Best	380	800	110	125	18	67
LOF	290	1200	150	50	18	67
Keyhole	290	800	70	125	18	67
Soot	290	1200	70	50	18	90

**Table 4:** Each build was designed to produce hundreds of SS-J3 tensile specimens which capture representative process variation expected during L-PBF manufacturing. The number of samples and the variable process conditions experienced by the samples for each build are summarized below.

Build ID	Number of Extracted Samples	Varied Location within the Build Volume	Varied Local Part Geometry	Varied Laser Module	Varied Laser Raster Process Parameters	Overhang Angle Relative to the *z*-Axis	Included Soot Generating Parts	Argon Flow Rate Setpoint (m^3^/h)	Powder Dosing Factor (%)	Used a Damaged Recoater Blade	Contours Enabled
B1.1	503	X	X	X		0°		40	200		X
B1.2	2705	X	X		X	0°		40	200		
B1.3	813	X	X	X	X	30°		40	200		X
B1.4	694	X	X	X		0°	X	25–40	200		X
B1.5	1584	X	X	X		0°		40	5–200	X	X

**Table 5:** Literature values compared to the range of measured YS, UTS, UE, and TE values observed across all the samples.

Source	YS (MPa)	UTS (MPa)	UE (%)	TE (%)
ASTM A240 [73]	170	480	40	N/A
Wrought [64]	261	562	66.0	72.8
B1.1–B1.5	70–420	80–610	0–69	4–94

**Table 6:** VPPM perceptron architecture. Columns indicate the type of network layer, the number of input channels (*C_i_*), and the number of output channels (*C_o_*). The variable $n_{feats}$ represents the number of elements in the super-voxel feature vectors and is fixed at 21 for this work.

Layer	*C_i_*	*C_o_*
Fully connected [75]	$n_{feats}$	128
10% dropout [75]	128	128
Fully connected	128	1

**Table 7:** The mean and standard deviations of the 5-fold validation RMS errors for the full-featured VPPM compared naïve predictions and the estimated intrinsic tensile measurement error. All table entries are equivalent to RMS errors.

	YS (MPa)	UTS (MPa)	UE (%)	TE (%)
Full-featured VPPM	24.7 ± 1.0	38.3 ± 0.9	9.0 ± 0.3	11.9 ± 0.1
Naïve predictions	35.4 ± 1.4	74.2 ± 1.8	16.1 ± 0.4	19.7 ± 0.2
Reduction from naïve	10.7	38.9	7.1	7.8
Measurement error	16.6	15.6	1.7	2.9

**Table 8:** Mean and standard deviations of the 5-fold validation RMS errors for each trained VPPM under each set of ablated training conditions. The first row of Table 7, indicating the final VPPM performance, is duplicated here for ease of reference.

	Fraction	YS (MPa)	UTS (MPa)	UE (%)	TE (%)
Full-featured VPPM	1.0	24.7 ± 1.0	38.3 ± 0.9	9.0 ± 0.3	11.9 ± 0.1
CAD and scan path	1.0	35.4 ± 1.5	76.3 ± 2.1	16.9 ± 0.5	20.1 ± 0.2
Printer log file	1.0	34.4 ± 1.5	73.5 ± 1.9	16.8 ± 0.5	19.8 ± 0.2
DSCNN classifications	1.0	25.7 ± 1.0	40.6 ± 0.8	9.0 ± 0.3	12.2 ± 0.2
Full-featured VPPM	0.2	24.7 ± 1.0	38.4 ± 0.9	9.1 ± 0.4	12.2 ± 0.2

**Figure A1:**
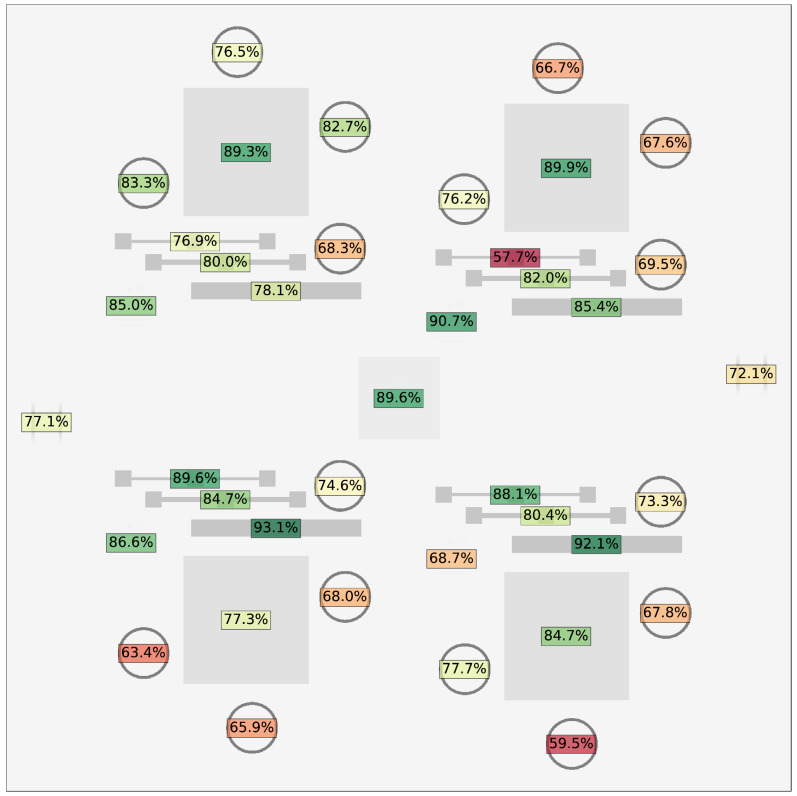
The volume percentage of each part from B1.1 that is classified as printed material by the DSCNN. Parts with lower percentages of printed material are shaded red, and parts with higher percentages are shaded green. Significant variation in this metric is present despite uniform process parameters.

**Figure A2:**
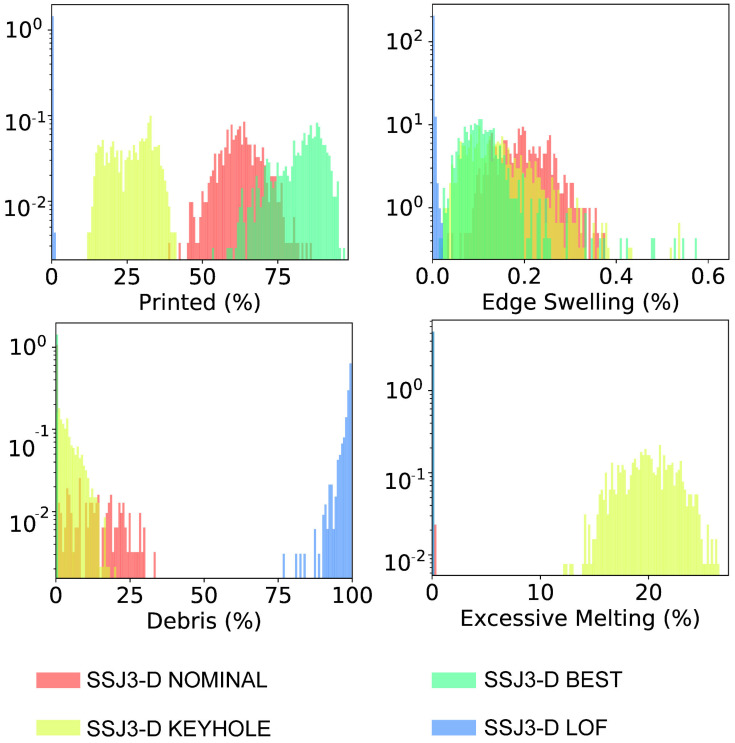
Histograms showing the distribution of four DSCNN classes throughout the volumes of the four SSJ3-D parts printed in B1.2. The *y*-axes are a log scaling of the bin density. These histograms represent data from layer 25 (1.25 mm) through layer 819 (40.95 mm). The four process parameter sets result in significant shifts in several of the distributions. For reference, one layer of the DSCNN results for this build is shown in Figure 5.

**Figure A3:**
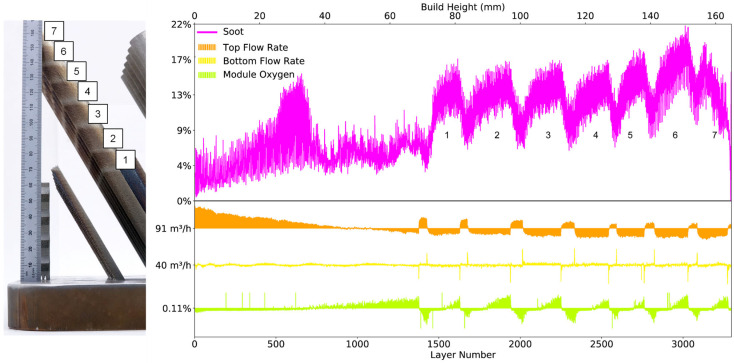
On the left, a picture of B1.3 taken after it was removed from the build chamber. Note the seven distinct numbered regions of increased oxidation on several of the parts. On the right, DSCNN soot segmentations (upper) and gas flow metrics (lower) are plotted as functions of layer height. Soot percentages are normalized by the cross-sectional area of the CAD geometry at each layer. Values from the log file are presented as deviations from their means, which are listed along the *y*-axis.

**Figure A4:**
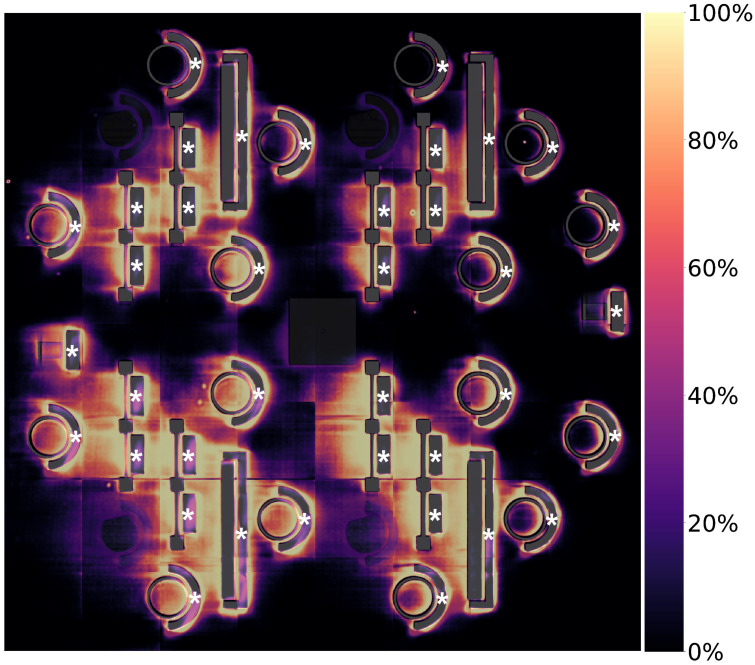
Projection of DSCNN soot segmentations from layer 0 through layer 1599 (79.95 mm) of build B1.4. The brightest regions indicate that soot was identified in 100% of the layers, and the darkest regions indicate that no soot was identified in that location for any of the layers. The shielding gas flows from right to left. White asterisks indicate the parts printed with the SOOT parameters; the remaining parts were printed with nominal parameters, as indicated in Figure 8. Tiling artifacts [28] and minor spotting on the camera’s viewport are also apparent in this visualization.

**Figure A5:**
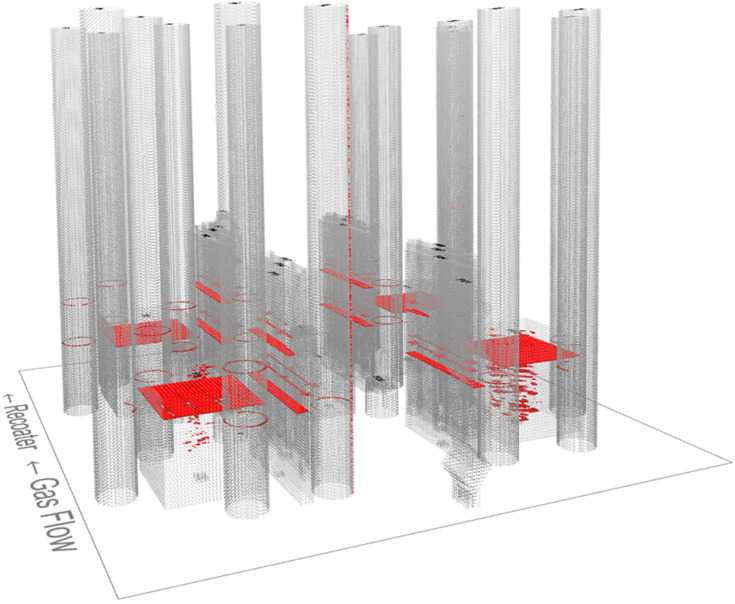
3D rendering of B1.5, with part CAD geometries shown in gray and DSCNN super-elevation segmentations shown in red. Super-elevation is primarily observed at the two build heights (33.05 and 52.00 mm), where artificial powder short feeds were induced. DSCNN segmentations of super-elevation are highlighted in red.

## Data Availability

The in situ powder imaging data, machine health sensor data, laser scan paths, tensile test results, and all associated metadata used in this work can be downloaded from https://doi.ccs.ornl.gov/ui/doi/452 (released on 28 September 2023). Representative training data for the DSCNN can be downloaded from https://doi.ccs.ornl.gov/ui/doi/341 (released on 23 April 2021) and https://doi.ccs.ornl.gov/ui/doi/417 (released on 13 February 2023).

## Appendix A

Because the resolution of the in situ sensor data is on the order of 100 µm, as are many of the void-type flaws [87] and surface features [65] expected to impact the tensile properties, a relatively small gauge section was expected to facilitate correlation of the ground truth measurements to in situ sensor signatures. Additionally, smaller specimen sizes allowed samples to be extracted from a more diverse set of part geometries, particularly thin-walled structures with thicknesses relevant to the nuclear power industry. However, inherent uncertainties in the spatial registration of the gauge sections to the in situ data, as well as physical challenges associated with performing tensile tests on extremely small specimens, ultimately limit the minimum size of the gauge section. Given these considerations, a standard sub-size specimen was selected for mechanical testing. Existing literature indicates that tensile properties measured using sub-size specimens may have higher variances and may not be fully indicative of bulk properties for L-PBF components, generally providing a conservative estimate of the bulk tensile properties [65]. Therefore, additional corrections may be required when applying the local property predictions at N7 to a full part; this topic is addressed in the Discussion section but is broadly beyond the scope of this work. Finally, it was advantageous for parallel research efforts that the tensile specimens maintain a form factor that is compatible with the standard canisters used for irradiation studies at ORNL’s High Flux Isotope Reactor (HFIR). Therefore, an SS-J3 geometry was ultimately selected, with nominal dimensions specified by ORNL’s generic metal irradiation specimen standard [69].

## Appendix B

Build B1.1, presented in Figure A1, shows the volume percentage of each part that was classified as printed material by the DSCNN. While all parts were printed with the nominal process parameters, the DSCNN classifications varied substantially across the print bed and the part geometries. One objective of this work is to determine the correlation between the sensor variation observed in a nominally processed build and the measured static tensile properties.

Each SSJ3-D part in build B1.2 was printed with a different parameter set. Figure A2 visualizes the layer-wise distributions of the printed, edge swelling, debris, and excessive melting DSCNN classes throughout the volumes of these four SSJ3-D parts. For the histogram of each part, the frequency of a class within a print layer was calculated as a percentage of the cross-sectional area of the CAD geometry at that layer. These results were then grouped such that layers with similar class frequencies were placed in the same bin. Parts with similar in situ sensor (visible-light camera) signatures have overlapping distributions, while parts with different signatures show differentiation between the distributions for one or more DSCNN classes. The four process parameter sets used to manufacture each of these SSJ3-D parts resulted in significant shifts in the anomalies segmented by the DSCNN. As expected, the LOF and keyhole SSJ3-D parts show the highest percentages of the debris and excessive melting classes, respectively. The SSJ3-D part printed with the best parameters has the highest percentage of printed material, whereas the edge swelling distributions are similar for the nominal, best, and keyhole SSJ3-D parts.

Figure A3 shows a post-build picture of several overhanging B1.3 parts with noticeable differences in surface oxidation, a plot of DSCNN soot classifications, and a plot of several shield gas metrics as functions of build height. Note that the seven instances of increased soot segmentation correlate with the seven distinct discoloration bands in the as-built parts. The authors hypothesize that as the oxygen concentration within the build chamber increased, oxidation of the spatter particles increased [88]. The darker spatter particles were easier for the DSCNN to segment as soot because of the increased contrast within the layer-wise images. When the oxygen concentration exceeded a threshold of approximately 0.18%, the printer automatically increased the argon gas flow rate across the top of the build chamber from 90 m^3^/h to 93 m^3^/h until the oxygen level returned to below 0.04%. At this point, the gas flow returned to its nominal setpoint, and the oxygen level was again allowed to climb, a pattern that resulted in the observed periodic behavior.

Figure A4 shows a heat map of DSCNN soot segmentations projected through the height of B1.4 onto the *x*-*y* plane, with brighter regions indicating higher percentages of soot. This visualization conveys the correlation between the right-to-left gas flow and the deposition of spatter particles across the print area. It is also apparent that, as intended, parts printed with the soot parameters (indicated by white asterisks) deposited significant amounts of spatter on top of the SSJ3-A, SSJ3-B, and SSJ3-C parts.

Figure A5 shows a 3D rendering of build B1.5, with DSCNN segmentations of super-elevation highlighted in red. The two artificially induced powder short feeds were detected as multiple consecutive layers of super-elevation by the DSCNN. Considering powder consolidation effects and typical powder packing densities [89], a typical powder layer is expected to be approximately 70 µm thick, whereas these anomalous layers are expected to result in powder layers up to 170 µm thick, depending on the location within the print area.

## Appendix C

Because of a focus on nuclear power applications, the maximum soak temperature was specified to retain the excellent as-built creep properties of the L-PBF-processed SS 316L material, as identified by Li et al. [90]. Soak times ranging from 0.5 to 24 h were then tested to minimize the total geometric distortion. The final furnace profile is specified in Table A1. To ensure that the larger SSJ3 parts reached the target soak temperature for the specified duration, a thermocouple was inserted into a 1-inch printed cube located in the middle of each build plate. All heat treatments were performed in ambient air.

**Table A1:** The post-build heat treatment profile applied to all five builds. The profile was designed to reduce part distortions due to residual thermal stresses while maintaining creep behavior appropriate for nuclear power applications.

Segment	Description
1	Ramp up at 10 °C/min to 650 ± 10 °C
2	Soak at 650 ± 10 °C for 24 ± 0.5 h
3	Furnace cool to 100 ± 20 °C

## Appendix D

**Table A2:** Set of features calculated based on part CAD geometries and the minimum and maximum values observed for the combined training and validation sets.

Feature		Min	Max	Justification and Description
1	Distance from edge (pixels)	1.115	27.000	Thermal histories [46] and porosity populations [58] for a volume are influenced by its distance from the part edge. Additionally, the surface roughness [9] of the SS-J3 samples is highly dependent upon whether it was extracted from the surface or the bulk of its parent part. To represent the planar distance of a super-voxel from a part edge, a distance transform [62] is applied to the CAD geometry with values allowed to saturate above 3.0 mm. A Gaussian blur with a 1.0 mm kernel and a standard deviation of 0.5 mm is then applied to mitigate computational edge effects, and a planar maximum is then performed within each super-voxel.
2	Distance from overhang (layers)	26.305	71.000	Thermal histories [91,92] for a volume are influenced by its distance above an overhanging surface. This distance is calculated for a vertical column of pixels with values allowed to saturate above 71 layers. A Gaussian blur with a 1.0 mm kernel and a standard deviation of 0.5 mm is then applied to mitigate computational edge effects.
3	Build height (mm)	8.875	72.775	Thermal histories for a volume can be influenced by its vertical distance away from the build plate [93]. This feature is calculated using the nominal print layer thickness and the known layer number.

**Table A3:** Set of features calculated based on DSCNN classifications of post-melt and post-spreading visible-light image data and the minimum and maximum values observed for the combined training and validation sets.

Feature		Min	Max	Justification and Description
4	Powder	0.000	0.632	Descriptions of DSCNN classes and their corresponding physical mechanisms are provided in Section 2.3. Each feature is calculated by applying a Gaussian blur with a 1.0 mm kernel and a standard deviation of 0.5 mm to the binary mask of each class. The resultant values represent the distance-weighted fractions of the surrounding area belonging to each class. The Gaussian kernel also facilitates the encoding of soot, which is most easily segmented on the powder surrounding a part.
5	Printed	0.000	1.000	
6	Recoater streaking	0.000	0.984	
7	Edge swelling	0.000	0.393	
8	Debris	0.000	1.000	
9	Super-elevation	0.000	0.033	
10	Soot	0.000	0.945	
11	Excessive melting	0.000	0.955	

**Table A4:** Set of features calculated from the sensor values recorded in the printer log file and the minimum and maximum values observed for the combined training and validation sets.

Feature		Min	Max	Justification and Description
12	Layer print time (s)	45.3	155.9	The selected log file variables encode sensor values which may be correlated to differences in thermal history (features 12, 16, and 17) [94], part oxidation (features 13, 14, 15, and 18) [88], and laser attenuation (features 13, 14, and 18) [95,96]. For each sensor, all the values recorded during a layer are averaged together, weighted by the temporal persistence of each individual sensor reading.
13	Top gas flow Rate (m^3^/h)	62.5	99.8	
14	Bottom gas flow rate (m^3^/h)	24.9	40.1	
15	Module oxygen (%)	0.000	0.148	
16	Build plate temperature (°C)	27	39	
17	Bottom flow temperature (°C)	41	60	
18	Actual ventilator flow rate (m^3^/h)	25.0	40.0	

**Table A5:** Set of features calculated from the laser scan path data and the minimum and maximum values observed for the combined training and validation sets.

Feature		Min	Max	Justification and Description
19	Laser module	0.000	1.000	Despite OEM calibration, the laser beam diameter and laser power may not behave identically between the two laser modules, potentially resulting in differences in thermal history, as well as melt pool morphology and size [97]. This feature encodes the laser module used to melt a given super-voxel.
20	Laser return delay	0.020	0.750	The amount of time between adjacent laser passes can influence the thermal history and the melt pool morphology and size [46]. To calculate a proxy for this metric, minimum and maximum filters with 1.0 mm kernels are applied to maps of the melt time since the start of each layer, and their pixel-wise difference is then calculated. A saturation value was chosen empirically to prevent saturation (excluding the stripe boundaries) for all the scan strategies used across the five builds.
21	Laser stripe boundaries	0.018	9.940	Thermal histories, porosity populations, and melt pool morphologies and sizes may be different at the interfaces between laser stripes [46]. To encode the locations of stripe boundaries, Sobel filters [47] were applied along both image axes to maps of the melt time since the start of each layer. The results are combined into a single pixel-wise value using the root mean square sum of the two Sobel filter responses.
